# IpsA, a novel LacI-type regulator, is required for inositol-derived lipid formation in *Corynebacteria* and *Mycobacteria*

**DOI:** 10.1186/1741-7007-11-122

**Published:** 2013-12-30

**Authors:** Meike Baumgart, Kerstin Luder, Shipra Grover, Cornelia Gätgens, Gurdyal S Besra, Julia Frunzke

**Affiliations:** 1 Institut für Bio- und Geowissenschaften, IBG-1: Biotechnologie, Forschungszentrum Jülich, 52425 Jülich, Germany; 2 School of Biosciences, University of Birmingham, Edgbaston, Birmingham, B15 2TT, UK

**Keywords:** Transcriptional regulator, *Corynebacterium glutamicum*, *Corynebacterium diphtheriae*, *Mycobacterium tuberculosis*, Cell wall synthesis, Mycolic acids, Mycothiol, Inositol, LM, LAM

## Abstract

**Background:**

The development of new drugs against tuberculosis and diphtheria is focused on disrupting the biogenesis of the cell wall, the unique architecture of which confers resistance against current therapies. The enzymatic pathways involved in the synthesis of the cell wall by these pathogens are well understood, but the underlying regulatory mechanisms are largely unknown.

**Results:**

Here, we characterize IpsA, a LacI-type transcriptional regulator conserved among *Mycobacteria* and *Corynebacteria* that plays a role in the regulation of cell wall biogenesis. IpsA triggers *myo*-inositol formation by activating *ino1*, which encodes inositol phosphate synthase. An *ipsA* deletion mutant of *Corynebacterium glutamicum* cultured on glucose displayed significantly impaired growth and presented an elongated cell morphology. Further studies revealed the absence of inositol-derived lipids in the cell wall and a complete loss of mycothiol biosynthesis. The phenotype of the *C. glutamicum ipsA* deletion mutant was complemented to different extend by homologs from *Corynebacterium diphtheriae* (dip1969) and *Mycobacterium tuberculosis* (rv3575), indicating the conserved function of IpsA in the pathogenic species. Additional targets of IpsA with putative functions in cell wall biogenesis were identified and IpsA was shown to bind to a conserved palindromic motif within the corresponding promoter regions. *Myo*-inositol was identified as an effector of IpsA, causing the dissociation of the IpsA-DNA complex *in vitro*.

**Conclusions:**

This characterization of IpsA function and of its regulon sheds light on the complex transcriptional control of cell wall biogenesis in the mycolata taxon and generates novel targets for drug development.

## Background

The human pathogens *Mycobacterium tuberculosis* and *Corynebacterium diphtheriae,* as well as many related species of the order *Corynebacteriales,* share a distinctive cell wall architecture that plays an important role in virulence and imparts effective protection against harsh environmental conditions and resistance to therapies [1]. One unique feature of these cell walls is the presence of long-chain α-alkyl, β-hydroxy fatty acids, called mycolic acids, which form a second bilayer close to the cell surface that is similar to the outer membrane of Gram-negative bacteria [2]. The enzymes and regulatory mechanisms involved in biosynthesis of the cell walls are primary targets for the development of new drugs.

**Figure 1:**
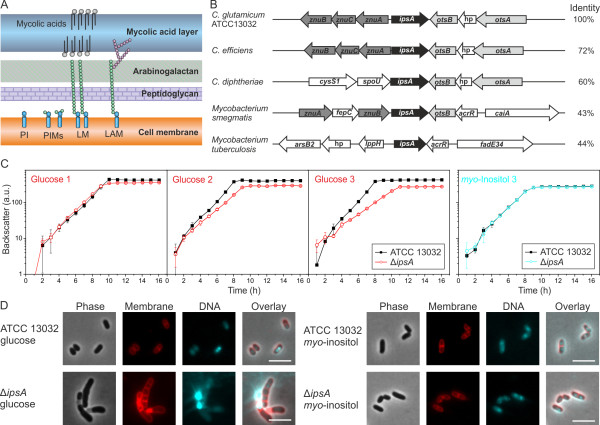
**IpsA plays an essential role in the integrity of the cell wall of *Corynebacteriales*. (A)** Current model of the structure of the cell wall of *Corynebacteria* and *Mycobacteria*. **(B)** Comparison of the organization of the IpsA genome locus in *C. glutamicum* and related species. IpsA and homologous proteins are shown in black. Data were taken from [5,6]. The percentage identity of the amino acid sequences to IpsA from *C. glutamicum* was taken from NCBI Blast. hp, hypothetical protein. **(C, D)** Growth curves **(C)** and phenotype **(D)** of *C. glutamicum* wild-type and Δ*ipsA*. Results in **(C)** show the growth (backscatter) from sequential cultures in minimal medium with glucose or *myo*-inositol as carbon source. After reaching the stationary phase, the cells were diluted into fresh medium at an initial OD_600_ of 1. Shown are three glucose cultures and the third *myo*-inositol culture (n = 3). In **(D)**, DNA was stained with 4’,6-diamidino-2-phenylindole (DAPI) (cyan) and lipophilic regions with nile red (red). The scale bar is 5 μm.

A great deal is known about these bacterial cell walls (Figure 1A). The plasma membranes are rich in phospholipids, such as phosphatidylglycerol, diphosphatidylglycerol and phosphatidylinositol (PI). While inositol is an essential polyol in eukaryotes, in eubacteria it is present only in the order *Corynebacteriales* where it serves both as a cell wall component and as a building block of mycothiol (MSH), the major antioxidant in *Mycobacteria*, similar to glutathione [3]. One function of PI is to anchor cell wall lipoglycans, specifically PI mannosides (PIMs), lipomannan (LM) and liporarabinomannan (LAM), to the plasma membrane [1,4]. The biogenesis of LAM is performed in consecutive steps in the following order: PI→PIM→LM→LAM.

In *Mycobacteria*, there is a three-step process for synthesis of PI. Step 1 is cyclization of glucose-6-phosphate to 1D-*myo*-inositol-3-phosphate, a reaction that is catalyzed by a *myo*-inositol phosphate synthase, Ino1 (Rv0046c) [7]. In step 2, the 1D-*myo-*inositol-3-phosphate is dephosphorylated by inositol monophosphatase (IMP) [8] to yield *myo*-inositol. Step 3 is the transfer of diacylglycerol (DAG) from cytidine diphosphate-diacylglycerol (CDP-DAG) to *myo-*inositol by a PI synthase, PgsA (Rv2612c) [9]. The enzymes Ino1 and PgsA are essential for the viability of *Mycobacteria*[10,11]. An *ino1* mutant of *M. tuberculosis* grows *in vitro* only in the presence of high inositol concentrations and was no longer able to kill SCID mice (see Movahedzadeh *et al*., [10]). In *Mycobacterium smegmatis*, *ino1* has been shown to be essential for growth in the absence of exogenous inositol [12]. Ino1 has therefore been proposed as a target for the development of new antibiotics [10,13].

In contrast to the wealth of knowledge about the biogenesis of cell walls, little is known about the regulatory circuits that control these processes [4]. IpsA was chosen as target for further studies from a set of mutants strains as a Δ*ipsA* mutant displayed a drastically altered cell morphology hinting towards a function in the control of cell wall biosynthesis. The catabolism of *myo*-inositol has been examined in *C. glutamicum*[14,15] but regulation of *myo*-inositol formation has not been deeply investigated up until now. Here, a regulator of inositol synthesis in the model organism *C. glutamicum* is described. Named IpsA, it is a novel LacI-type regulator that acts as an inositol-dependent transcriptional activator of the *myo*-inositol phosphate synthase gene. IpsA function is conserved throughout *Corynebacteria* and *Mycobacteria*, including the prominent pathogenic species *C. diphtheriae* and *M. tuberculosis*.

## Results

### IpsA is a novel LacI-type regulator conserved in the *Corynebacteriales*

IpsA was assigned by homology to the LacI family of transcriptional regulators, which often coordinate the available nutrients with the expression of catabolic genes [16]. The corresponding genomic loci display a remarkable level of conservation across the *Corynebacteriales* (Figure 1B). In previous studies, the structure of IpsA (Cg2910) was analyzed (PDB:3H5T); its physiological function is the subject of this study.

### IpsA deletion affects cell shape and growth

To gain insight into the function and possible target genes of IpsA, an in-frame deletion mutant was constructed. Growth rates and final backscatter of Δ*ipsA* cells were significantly below control levels using minimal medium with glucose as carbon source, and these shortfalls increased in magnitude with successive subcultivation (Figure 1C). The morphology of the mutant strain was altered, as revealed by fluorescence microscopy with staining of DNA (4’,6-diamidino-2-phenylindole (DAPI)) and membranes (nile red). Δ*ipsA* cells formed chain-like structures and failed to divide properly. DNA in the cells was unevenly distributed and was frequently found in the extracellular space, indicative of severe damage to the cell membrane and/or the cell wall (Figure 1D, for a larger sample see Additional file 1: Figure S1). Membrane staining revealed several intact cell septa in the unshaped mutant cells.

**Figure 2:**
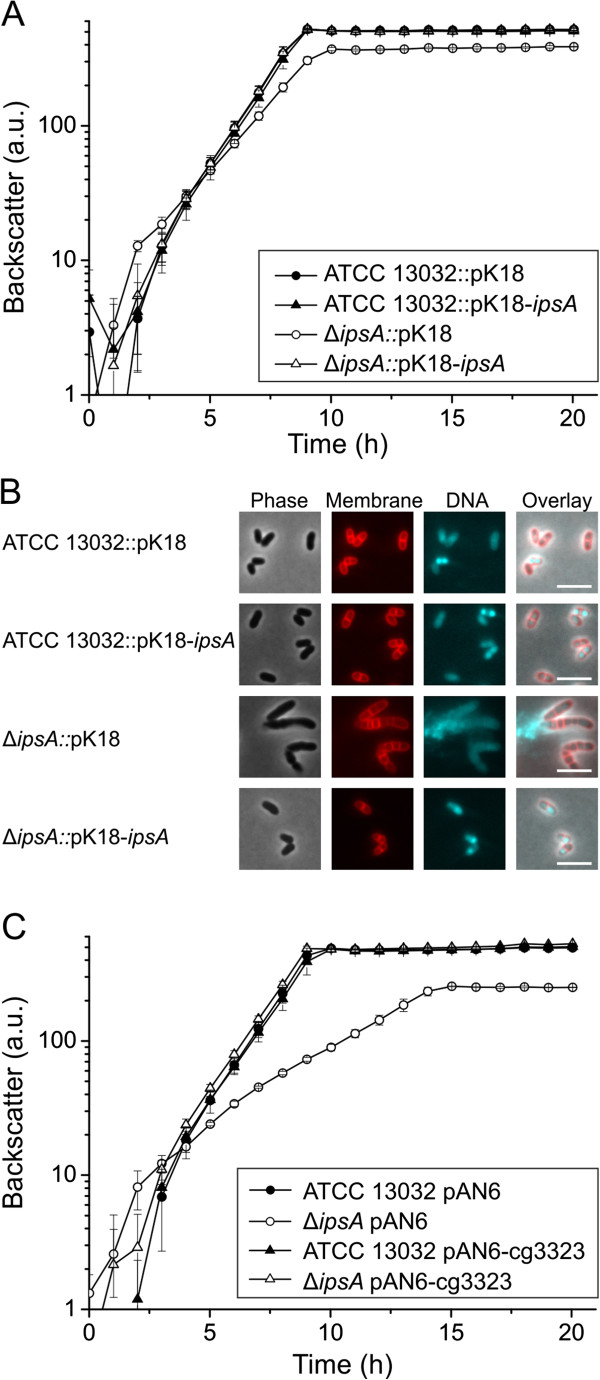
**Complementation of the Δ*ipsA *phenotype with *ipsA *and the target gene *ino1 *(cg3323)*.*** For chromosomal complementation, the gene *ipsA* was integrated into the intergenic region of cg1121 to cg1122 under control of the native promoter. **(A)** Growth of the strains in CGXII minimal medium with 2% (w/v) glucose as carbon source. The average and standard deviation of three biological replicates is shown. **(B)** Fluorescence microscopy images of stationary phase cells of the cultures presented in (A). **(C)** Plasmid-based complementation with the target gene *ino1* (cg3323). The strains were cultivated in CGXII minimal medium with 2% (w/v) glucose and 50 μM isopropyl β-d-1-thiogalactopyranoside (IPTG) for induction of *ino1* expression.

Both the growth defect and altered morphology could be partially overcome by plasmid-encoded *ipsA* under control of the native or an inducible promoter (data not shown). These efforts revealed that increased levels of IpsA also affect growth of *C. glutamicum*. Full restoration of the wild-type phenotype was only achieved when *ipsA* was reintegrated into the mutant strain (intergenic region cg1121 to cg1122) and expressed from its native promoter (Figure 2A, B). When the cells were cultivated with *myo*-inositol instead of glucose as carbon source, no differences were observed between the wild-type and Δ*ipsA* strains in terms of growth and morphology (Figure 1C, D).

### The impact of *ipsA* mutation on transcription

To elucidate the transcriptional changes caused by the deletion of *ipsA*, DNA microarray experiments were performed. More than 450 genes showed alterations in mRNA level greater than a factor of 2, using cells in the exponential growth phase (OD_600_ = 5) cultivated in minimal medium with glucose as carbon source (Additional file 1: Table S3, full data deposited in GEO database [17] under accession number GSE50210).

The expression of the gene cg3323, which encodes a *myo*-inositol phosphate synthase (*ino1)*, and of the operon (cg0044 to cg0046), which encodes an uncharacterized ABC transporter, was reduced by more than a factor of ten in the mutant strain (Table 1). Other downregulated genes included a flavin-containing monooxygenase (cg3195), a hypothetical endoglucanase (cg2896), and two further transporters (cg0621 to cg0623 and cg2181 to cg2184) (Additional file 1: Table S3). Among the strongly upregulated genes was a cluster encoding enzymes for inositol catabolism (cg3389 to cg3392), an operon encoding both *menE* and a putative integral membrane protein (cg0533 to cg0534), and a putative dinucleotide-binding enzyme (cg1421). Many of the affected genes are components of the DtxR stimulon, which plays a central role in iron homeostasis in *C. glutamicum*[18,19]. We speculate that these changes are the result of an impaired iron uptake of the mutant strain due to the defects in the cell wall. In combination with the observed growth phenotype of the mutant strain, the alterations in gene expression indicate that IpsA is a major regulator of inositol-derived cell wall components.

**Table 1:** Transcriptome analysis of an IpsA deletion mutant

**Locus tag**	**Gene**	**Annotated function**	**Ratio**
cg3323	*ino1*	*Myo*-inositol phosphate synthase	0.047
cg0044	*rbsB*	ABC transporter/periplasmic d-ribose-binding protein	0.082
cg0045	*-*	Probable ABC transport protein, membrane component	0.138
cg2910	*-*	Transcriptional regulator, LacI family	0.173
cg3195	*-*	Flavin-containing monooxygenase	0.178
cg0046	*-*	Probable ABC transport protein, ATP-binding component	0.323
cg3210	*-*	Cell envelope-related transcriptional regulator	0.411
cg1421	*-*	Putative dinucleotide-binding enzyme	5.214
cg0533	*menE*	*O*-succinylbenzoic acid-CoA ligase	5.467
cg0534	*-*	Putative integral membrane protein	5.597

mRNA ratios (Δ*ipsA*/wild-type) of IpsA target genes in microarray experiments are shown. Presented are all genes where binding of IpsA could be shown in electrophoretic mobility shift assays (EMSAs) as well as IpsA itself, confirming the deletion.

### Identification of direct IpsA target promoters

**Figure 3:**
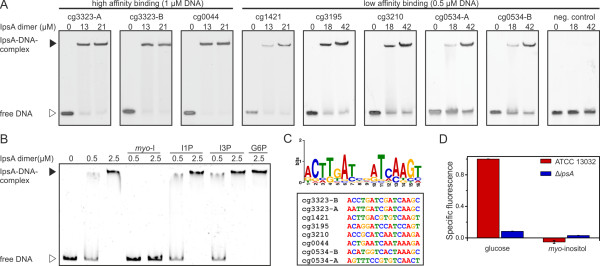
**Target genes, effector and binding sites of IpsA. (A)** Binding of IpsA to 30 bp oligonucleotides covering the binding sites in the putative target promoters. As negative control the oligonucleotide cg3323-d (sequence AGGTCTGATTTCTGCTGGGAATCCCCACAT) was used, which is located immediately downstream of the IpsA binding sites in the cg3323 promoter. **(B)** The influence of intermediates in inositol metabolism (5 mM each) on the binding of IpsA to the *ino1* promoter. *myo*-I, *myo*-inositol; I1P, 1D-*myo*-inositol-1-phosphate; I3P, 1D-*myo*-inositol-3-phosphate; G6P, glucose-6-phosphate. **(C)** The IpsA consensus motif predicted by MEME [20] and an overview of the sites in the corresponding promoter regions. **(D)** Promoter-fusion studies using the promoter of *ino1* fused to *eyfp* (pJC1-Pcg3323-eyfp). The specific fluorescence of ATCC 13032 containing this plasmid on glucose was set to 1 and the other values were calculated accordingly.

The microarray experiments revealed several putative target genes of IpsA. To determine whether these genes are directly regulated by IpsA, the promoter regions of the respective genes were tested in electrophoretic mobility shift assays (EMSAs) for the formation of complexes with purified IpsA protein. We analyzed the promoters of all genes that were more than fourfold regulated but excluded ribosomal proteins and genes belonging to the DtxR/RipA regulon. DNA fragments (approximately 500 bp) covering the promoter regions of putative target genes, were incubated with increasing IpsA-His concentrations. IpsA-His bound with high affinity to the promoter regions of cg0044 and *ino1* (cg3323) and with lower affinity to the promoter regions of cg0534 (a putative integral membrane protein), cg1421 (a putative dinucleotide binding enzyme), cg1476 (*thiC*, thiamine biosynthesis protein ThiC), cg1918 (a putative secreted protein), and cg3195 (flavin containing monooxygenase) (Figure 3; all tested fragments are shown in Additional file 1: Figure S3).

### Determination of the IpsA DNA binding site

**Figure 4:**
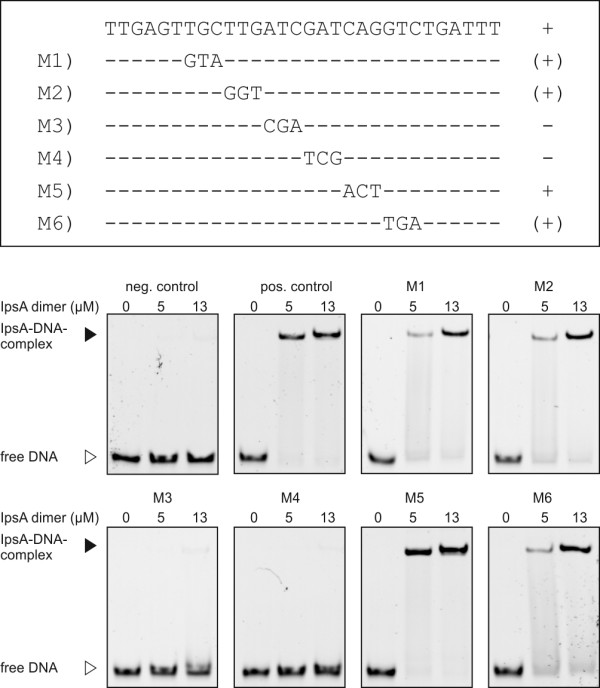
**Mutational analysis of one of the IpsA binding sites in the *ino1 *promoter.** The importance of the predicted DNA sequence motif for IpsA binding was tested in electrophoretic mobility shift assays (EMSAs) with DNA fragments in which three nucleotides of the proposed motif were exchanged, as indicated. A + indicates that the mutated fragment was bound with the same affinity as the unaltered wild-type fragment (positive control); (+) indicates that the mutated fragment was shifted, but with lower affinity; - indicates that the mutated fragment was not shifted. In the case of M3 and M4, binding was completely abolished by the mutation, indicating that the six central base pairs are crucial for IpsA binding. For M1, M2 and M6 a slight decrease of binding was observed. The binding of M5 was unchanged.

Using EMSAs, two separate binding sites were identified in the promoter of *ino1* and one in the promoter of cg0044 (Figure 3A and Additional file 1: Figure S4). Analysis of these sites using the motif-based sequence analysis tool MEME [20] revealed a conserved palindromic motif, which was subsequently verified by mutational analysis (Figure 4). In particular, the central six base pairs appear to be crucial for IpsA binding. The preliminary motif was used to search for further binding sites in putative target genes and across the genome of *C. glutamicum*. Binding sites were identified by EMSAs using oligonucleotides in the promoters of cg1421, cg3195, cg3210 and cg0534 (Table 1, Figure 3A and Additional file 1: Figure S5). The identified motifs were used to define the IpsA consensus motif shown in Figure 3C.

### *Myo*-inositol is an effector molecule of IpsA

The enzyme encoded by the main IpsA target gene is Ino1, a *myo*-inositol phosphate synthase that catalyzes the formation of 1D-*myo-*inositol-3-phosphate from glucose-6-phosphate [7]. We tested several metabolites involved in the relevant pathway for their influence on the formation of the IpsA-DNA complex. A clear backshift was observed for *myo*-inositol but not for 1D-*myo-*inositol-1-phosphate, 1D-*myo-*inositol-3-phosphate or glucose-6-phosphate (Figure 3B). The effect of *myo*-inositol could be observed at concentrations down to 0.2 mM (Additional file 1: Figure S6). This effect is specific for IpsA DNA binding, as the addition of up to 50 mM *myo*-inositol did not have any effect on the formation of an unrelated protein-DNA complex (for example, AcnR and the *acn* promoter, Additional file 1: Figure S7).

### IpsA is required for inositol-dependent regulation of *ino1*

The major function of IpsA revealed thus far is the activation of *ino1* in the absence of external inositol. Consistent with this, the growth and cell morphology of the Δ*ipsA* strain is complemented by the constitutive expression of *ino1* under standard conditions (Figure 2C). To study the influence of IpsA and *myo-*inositol on the expression of *ino1 in vivo*, we fused the *ino1* promoter to *eyfp* (enhanced yellow fluorescent protein) and monitored the fluorescence output of the wild-type and the Δ*ipsA* strain during growth on glucose and *myo*-inositol (Figure 3D). Strong promoter activity was observed in the wild-type during growth on glucose. Expression of *eyfp* was significantly decreased in the *ipsA* deletion strain and was lower still when either strain was cultivated with *myo*-inositol as the carbon source.

### IpsA function is conserved in *C. diphtheriae* and *M. tuberculosis*

**Figure 5:**
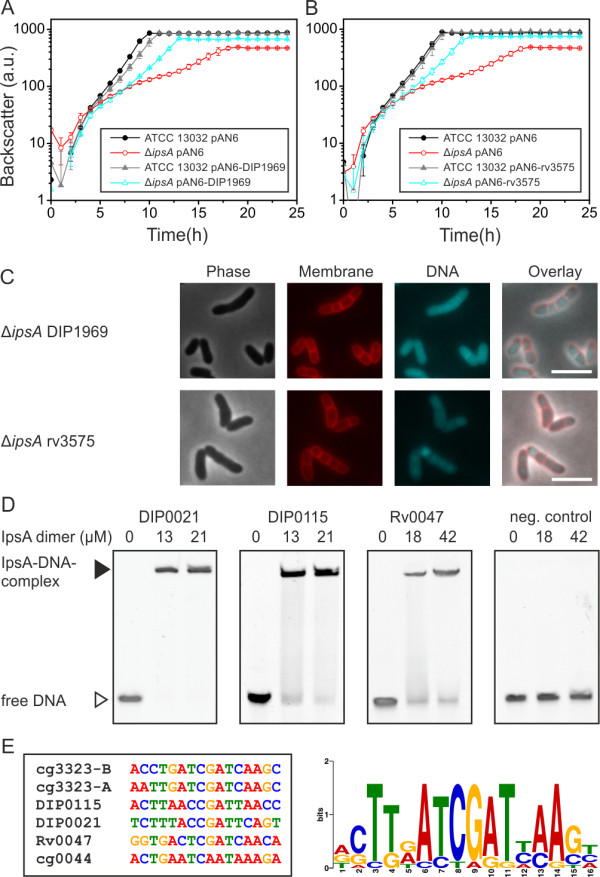
**IpsA in *Corynebacterium diphtheriae *and *Mycobacterium tuberculosis*.***Corynebacterium glutamicum* Δ*ipsA* carrying the plasmids pAN6, pAN6-DIP1969 or pAN6-rv3573 and *C. glutamicum* wild-type with pAN6 as control were cultivated in CGXII with glucose without isopropyl β-d-1-thiogalactopyranoside (IPTG) **(A)** or with 50 μM IPTG **(B)**. **(C)** Microscopic phenotypes of the complemented strains. DNA was stained with 4’,6-diamidino-2-phenylindole (DAPI) (cyan) and lipophilic regions with nile red (red), scale bar 5 μm. **(D)** Oligonucleotides (30 bp, 1 μM for DIP0021 and DIP0115, 0.5 μM for Rv0047 and the negative control) covering the predicted binding sites in the promoter regions of the respective genes were incubated with IpsA at the given concentrations and analyzed on 15% native polyacrylamide gels. In *M. tuberculosis*, Rv0046 is organized in an operon with Rv0047. A binding site was identified within the open reading frame (ORF) of Rv0047, suggesting the occurrence of a second promoter upstream of *ino1*. **(E)** IpsA binding motif derived from the high affinity *C. glutamicum* targets and the *C. diphtheriae* and *M. tuberculosis* binding sites.

The conservation of IpsA in the *Corynebacteriales* suggests that it may play a similar role in pathogenic relatives. To assess this possibility, we tested *in vivo* complementation of *C. glutamicum* Δ*ipsA* with the IpsA homologs of *M. tuberculosis* (Rv3575) and *C. diphtheriae* (DIP1969). Partial complementation of growth and morphology was achieved (Figure 5A-C). Growth was still slightly retarded but the final OD was significantly increased in comparison with the Δ*ipsA* strain. Likewise, the morphological phenotype of the complemented strains fell in between the wild-type and the Δ*ipsA* strain, with a slightly better complementation by DIP1969 compared to Rv3575. Most cells displayed the wild-type morphology and, while some chain-like cell clusters were observed (Figure 5C), these were shorter than clusters of the Δ*ipsA* strain (Figure 1D).

We also tested the binding of IpsA to the promoter regions of *C. diphtheriae* and *M. tuberculosis* homologs of cg3323 and cg0044 using EMSAs (Figure 5D). *C. glutamicum* IpsA bound strongly to the *C. diphtheriae* promoters DIP0115 and DIP0021 and the binding sites were determined (Figure 5E and Additional file 1: Figure S8). In *M. tuberculosis*, Rv0046c is organized in an operon with Rv0047c. A binding site was identified within the open reading frame (ORF) of Rv0047c, suggesting the occurrence of a second promoter upstream of *ino1* (Figure 5E).

### Mycothiol synthesis is abolished in *C. glutamicum ∆ipsA*

**Figure 6:**
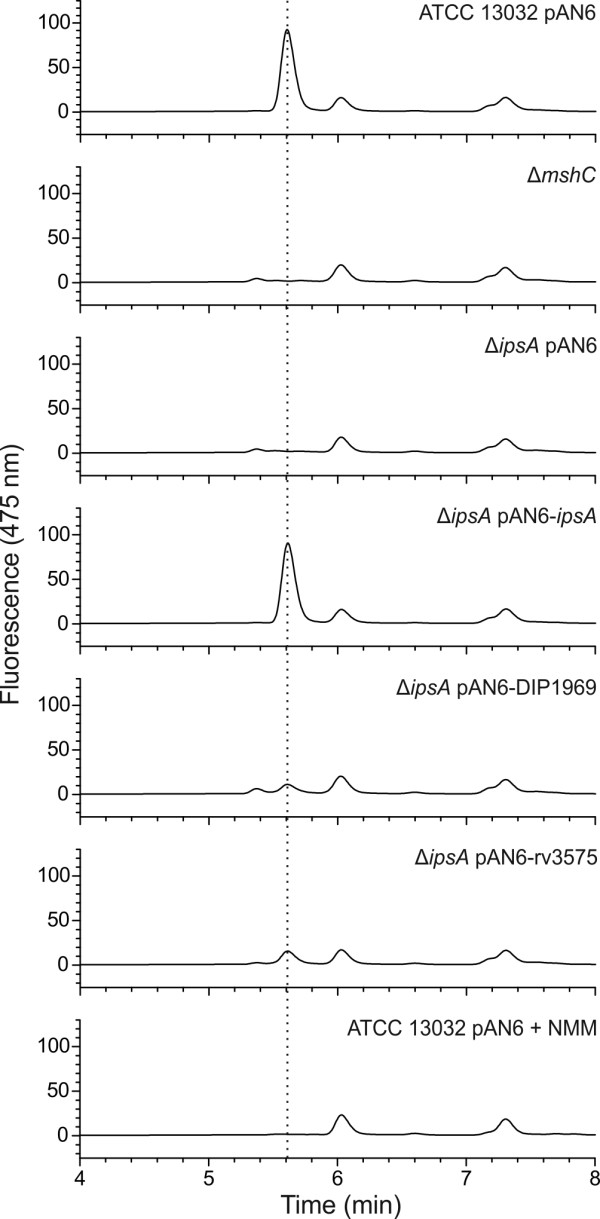
**Determination of mycothiol production in *Corynebacterium glutamicum *wild-type and different mutants and complemented strains.** The mycothiol was derivatized using bromobimane, separated by high-performance liquid chromatography (HPLC) and monitored using a fluorescence detector (390 nm excitation and 475 nm emission). As control, the mycothiol deficient strain Δ*mshC* was used as well as a wild-type sample that had been treated with *N*-methyl-maleimide (NMM) to block thiols prior to derivatization. The peak assumed to be mycothiol is marked with a dotted line. Presented is a representative chromatogram of three biological replicates each.

Besides being a precursor of PI and derived lipids, *myo*-inositol is one of the building blocks of mycothiol, the major antioxidant of *Corynebacteria* and *Mycobacteria*. Therefore, we measured mycothiol in a set of our strains (Figure 6) using a method previously published for *C. glutamicum*[21]. The mycothiol peak was identified by comparison of wild-type chromatograms with those of a mutant depleted in mycothiol synthesis (Δ*mshC*). To proof that the corresponding peak, lacking in Δ*mshC,* represents mycothiol, thiols were blocked with NMM (*N*-methyl-maleimide) prior to derivatization with bromobimane. In this sample, the largest peak of the wild-type at 5.6 minutes was indeed absent the two control samples. In fact, this mycothiol peak was absent in the Δ*ipsA* strain but could fully be restored by plasmid-based expression of *ipsA* (Figure 6). Interestingly, the mycothiol measurements showed a partial complementation with the *ipsA* homologs from *C. diphtheriae* and *M. tuberculosis*, which is in line with our previous findings regarding growth and morphology of cross-complemented strains (Figure 5).

### PI-based glycolipid synthesis is lost in *C. glutamicum ∆ipsA*

**Figure 7:**
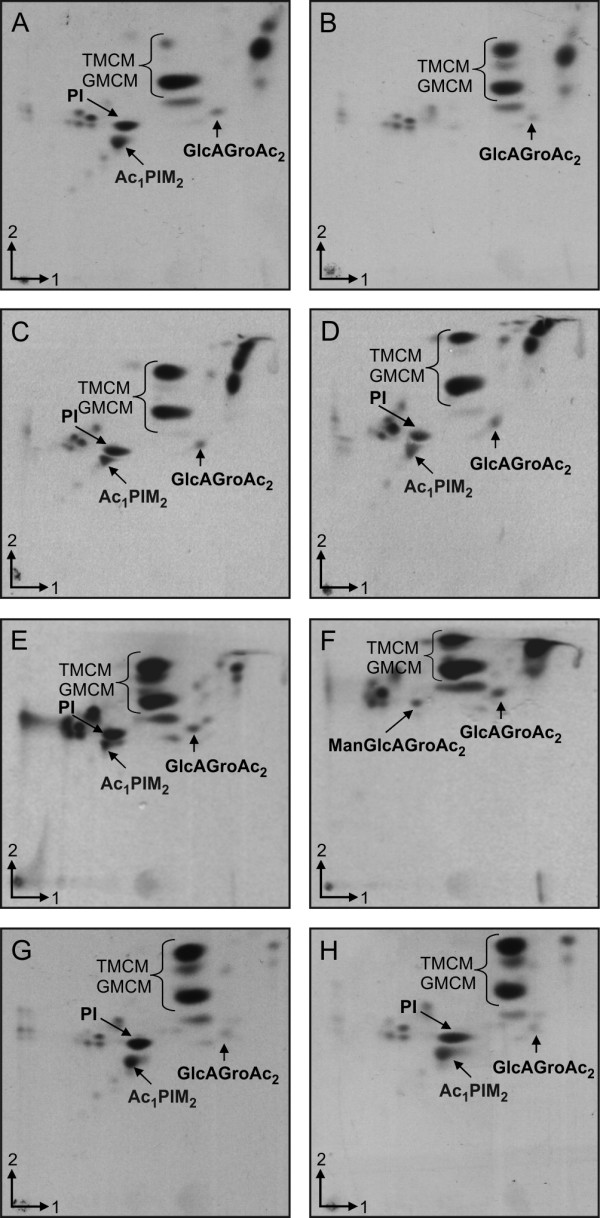
**Two-dimensional thin-layer chromatography (2D-TLC) analysis of [**^**14**^**C]-labeled polar lipids from *Corynebacterium glutamicum *cells grown in glucose (A-F) or *myo*-inositol (G-H). (A)** and **(G)***C. glutamicum* ATCC 13032, **(B)** and **(H)** ATCC 13032 Δ*ipsA,***(C)** ATCC 13032 Δ*ipsA* pAN6-cg3323*,***(D)** ATCC 13032 Δ*ipsA*::pK18int-ipsA*,***(E)** ATCC 13032 Δ*ipsA* pAN6-DIP1969 and **(F)** ATCC 13032 Δ*ipsA* pAN6-Rv3575. The cells were cultured in CGXII with either glucose **(A-F)** or *myo*-inositol **(G, H)**. PI, phosphatidylinositol; TMCM, trehalose monocorynomycolate; GMCM, glucose monocorynomycolate; Ac_1_PIM_2_, monoacylated phosphatidyl *myo*-inositol dimannoside; GlcAGroAc_2_, 1,2-di-*O*-C_16_/C_18:1_-(α-d-glucopyranosyluronic acid)-(1→3)-glycerol (GL-A); ManGlcAGroAc_2_ 1,2-di-*O-*C_16_/C_18:1_-(α-d-mannopyranosyl)-(1→4)-(α-d-glucopyranosyluronic acid)-(1→3)-glycerol (GL-X).

To study the effect of IpsA on the formation of PI-based phosphoglycolipids, [^14^C]-labeled polar lipids were extracted from wild-type and *∆ipsA C. glutamicum*. The glycolipid profiles were examined using two-dimensional thin-layer chromatography (2D-TLC) (Figure 7). The wild-type strain showed presence of AcPIM_2,_ PI, Gl-A (GlcAGroAc_2_) and Gl-X (ManGlcAGroAc_2_) in lipid extracts as compared to known standards and previous studies [22], isolated from the cells grown with either *myo*-inositol or glucose. Under normal conditions, *C. glutamicum* produces both PI and Gl-X, which is an alternative glycolipid anchor to PI for synthesis of Cg-LM. The glycolipid Gl-X is derived from Gl-A, its precursor product. Although both PI and Gl-X can be visualized on TLC analysis, the formation of Gl-X is distinguished only in the case of mutants lacking PI, as both Gl-X and PI do not separate out as distinct spots [23,24]. However, Gl-A appears as a distinct spot on the TLC results in both wild-type and mutant strains. For convenience, the TLC results for polar lipid extracts from the *ipsA* mutant defective in PI synthesis have been marked to depict Gl-X at the same position at which PI is depicted in the wild-type and complemented strains. The labeling for Gl-A remained unchanged. Interestingly, the complete absence of AcPIM_2_ was observed in lipid extracts isolated from *C. glutamicum ∆ipsA* when cultured in minimal media supplemented with glucose indicating the loss of PI-based glycolipid synthesis in the mutant (Figure 7B). The lipid profiles of complemented strains *C. glutamicum ΔipsA*::pK18-ipsA and *C. glutamicum* Δ*ipsA* harboring cg3323 and DIP1969 in plasmid pAN6, were identical to the wild-type when grown on glucose, demonstrating successful complementation of the loss of gene function in these strains (Figure 7C-E). However, the lipid extracts from *C. glutamicum* Δ*ipsA* harboring Rv3575 in plasmid pAN6 cultured under the same conditions displayed no complementation as none of the components was stained with phosphate stain (results not shown). The lipid extracts isolated from the mutant and complemented strains cultured in minimal media supplemented with *myo*-inositol were similar to wild-type, with distinct AcPIM_2_ and PI production (Figure 7G, H and Additional file 1: Figure S9).

### Analysis of lipoglycan extracts

Since the lipoglycans LM and LAM are built on PIMs which are mannosylated products of PI, the effect of IpsA was also studied on these lipoglycans extracted from the wild-type, *C. glutamicum ∆ipsA* and the complemented strains (Additional file 1: Figure S10A). LAM and Cg-LM-A/B were clearly present in extracts from the wild-type cultured in glucose or *myo*-inositol. However, the lipoglycan extracts from the mutant *C. glutamicum ∆ipsA* grown on glucose revealed no LAM while a band corresponding to Cg-LM-B could be observed. On glucose the complemented strains *C. glutamicum* Δ*ipsA*::pK18-ipsA and *C. glutamicum* Δ*ipsA* containing plasmid encoded Cg3323 and DIP1969 exhibited lipoglycan profiles identical to the wild-type as distinct bands corresponding to both LAM and Cg-LM-A/B. However, no band corresponding to LAM was observed in lipoglycan extracts of *C. glutamicum* Δ*ipsA* complemented with Rv3575c in pAN6 plasmid grown in minimal media supplemented with glucose. Instead, a band corresponding to Cg-LM-B was evident. The lipoglycan extracts isolated from the mutant and complemented strains cultured in minimal media supplemented with *myo*-inositol were similar to wild-type with distinct LM and LAM (Additional file 1: Figure S10B).

## Discussion

**Figure 8:**
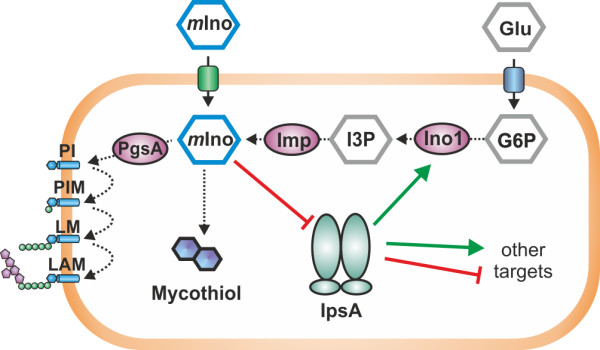
**Model of IpsA function.** In *Corynebacteria* and *Mycobacteria*, *myo*-inositol (*m*Ino) is an important building block for cell wall components and mycothiol. *m*Ino can be taken up from the culture medium or synthesized from glucose-6-phosphate (G6P) via 1D-*myo*-inositol-3-phosphate (I3P). When *m*Ino concentration is low, IpsA activates *ino1*, encoding *myo*-inositol phosphate synthase, which catalyzes the formation of I3P from G6P. Besides *ino1*, IpsA activates or represses several other targets of unknown function.

The metabolic pathways for biogenesis of the cell wall in *Mycobacteria* and *Corynebacteria* are well characterized, with few gaps remaining to be filled. In contrast, the regulation of these processes remains largely undiscovered. Here, we describe the novel transcriptional regulator IpsA, which functions as an activator of *ino1* and other target genes most likely involved in the synthesis of inositol-derived cell wall components and mycothiol (MSH) in *Corynebacteria* and *Mycobacteria* (Figure 8). *Myo*-inositol was shown to act as effector of IpsA and our studies are consistent with a conserved function for this regulator throughout the *Corynebacteriales*.

The polyol *myo*-inositol has an important function in *Mycobacteria* and *Corynebacteria*: it serves as the key building block for a large class of cell wall phospholipids, as well as for the antioxidant MSH [1,3]. Several studies have illustrated the potential of the underlying biosynthetic pathways as putative drug targets [13]. For example, an *ino1* mutant of *M. tuberculosis* can only grow in the presence of high concentrations of *myo*-inositol [10] and a *M. tuberculosis* mutant defective in MSH biosynthesis requires catalase for growth on plates [25]. Our studies have shown that the deletion of *ipsA* leads to a complete loss of mycothiol biosynthesis, demonstrating the importance of IpsA also for this metabolic pathway.

*Myo*-inositol and its phosphate derivatives are common substances in eukaryotes and are therefore present in significant quantities in the natural habitats of *M. tuberculosis*, *C. diphtheriae* (the human body) and *C. glutamicum* (soil) [26]. It has been shown that *myo*-inositol can be used by *C. glutamicum* as a carbon and energy source, and several transporters, enzymes and regulators of the catabolic pathway have been identified [14,15]. In the present study, microarray analysis revealed that the two main target genes of IpsA, *ino1* and cg0044, are strongly downregulated during growth on inositol compared to growth on glucose, which is in line with IpsA being an inositol-dependent activator of both genes/operons [14].

Bioinformatic analysis of *ipsA* and its homologs in other *Corynebacteria* and *Mycobacteria* revealed conservation of the gene among these species (Figure 1B). In many genomes of the *Corynebacteriales, ipsA* is located next to *znuACB*, which encodes a putative zinc transport system regulated by Zur [27]. Furthermore, genes involved in the biosynthesis of trehalose (*otsAB*), which is also an important component of the cell wall of the *Corynebacteriales,* are frequently located downstream of *ipsA*, but their orientations are divergent [28,29]. The product of the main target gene of IpsA, Ino1, contains a zinc ion in its catalytic center and needs NAD^+^ for its reaction [30].

The transcriptional organization of *ino1* differs between *Corynebacteria* and *Mycobacteria.* While *ino1* is the only gene in the transcriptional unit in *Corynebacteria*, it is cotranscribed with a preceded, uncharacterized PadR-like transcriptional regulator in *Mycobacteria*[31] (Additional file 1: Figure S11). Given the genomic organization, we suspect that this regulator is an additional player in the control of *ino1* expression.

That *ino1* is the main target gene of IpsA is supported by the observation that the *ipsA* phenotype was complemented by overexpression of *ino1*. One could assume that this is the only real target gene of IpsA, but it is not unlikely, that other target genes only appear to be important under different conditions, for example, another carbon source or certain stress conditions. Based on DNA Microarrays and EMSAs we identified several other direct target genes of IpsA, all of which were previously uncharacterized. The operon cg0044 to cg0046 encodes an ABC transporter of unknown function. It may be involved in the uptake of inositol(-derived) carbohydrates required for cell wall biogenesis. The product of cg3210 is annotated as a cell envelope-related transcriptional regulator of the LytR type. It has a transmembrane domain and is conserved in *Corynebacteria* and *Mycobacteria*. Cg1421 is a putative dinucleotide-binding enzyme that has some similarity with coenzyme F420-dependent NADP oxidoreductases. Cg3195 is annotated as a flavin-containing monooxygenase and has, like Cg1421, a NAD(P) binding domain. As many MSH-dependent detoxification reactions require NAD or NADP as cofactors [3], Cg1421 and Cg3195 may be involved in cofactor supply or regeneration for these processes. The operon cg0534 to cg0533 encodes a putative integral membrane protein of the YphA family and *menE*, an *O*-succinylbenzoic acid-CoA ligase. Although the functions of the target genes besides *ino1* are largely unknown, there are some clear links to cell wall synthesis and regulation, and likely also to MSH-associated reactions. Thus, our analysis of the IpsA regulon has revealed several novel and interesting targets for further studies.

Among the target genes of IpsA are not only activated but also repressed genes. The localization of the IpsA binding site in the particular promoter regions matches very well with the type of regulation (Additional file 1: Figure S5). For the activated genes (cg3323, cg0044, cg3210, and cg3195) IpsA binding sites are located upstream of the -35 region. In the promoters of the repressed genes (cg1421 and cg0534) the IpsA binding sites overlap with the -35 and/or the -10 region. This is a further indication that the altered mRNA level we see in the DNA microarrays is a direct effect of the IpsA deletion.

The structure-function relationship of IpsA is comparable to other members of the LacI family of transcriptional regulators. They all contain a DNA-binding domain at the N-terminus of the protein, composed of three α-helices that form a helix-turn-helix motif that binds to the major groove of the operator. The tetramerization of LacI takes place at the C-terminus, at residues 340 to 357, (Additional file 1: Figure S12) [32]. These residues are missing in IpsA, which is in agreement with our gel filtration analysis that indicated a dimeric conformation for IpsA. A computational analysis of many LacI regulators and binding sites revealed a highly conserved hydrophobic amino acid at position 54 (mostly leucine, L64 in IpsA), which inserts into a conserved, central CG group in the operator. This group is present in both binding motifs of the *ino1* promoter in *C. glutamicum*[33].

The formation of 1D-*myo-*inositol-3-phosphate from glucose-6-phosphate by Ino1 represents the first step of PI synthesis; it provides the scaffold on which cell wall components such as PIMs, LM and LAM are built. Our chromatographic analysis of radiolabelled polar lipid extracts of the mutant *C. glutamicum ∆ipsA* grown in minimal media with glucose generated a characteristic phenotype, namely that the mutant strain was deficient in AcPIM_2_ and PI while Gl-A and Gl-X were unaffected. In addition, the mutant strain grown on minimal media supplemented with glucose failed to produce PI-based LAM. Instead, the GL-X-based LM, Cg-LM-B was apparent on sodium dodecyl sulfate polyacrylamide gel electrophoresis (SDS-PAGE) analysis of lipoglycans, which could explain why *C. glutamicum ∆ipsA* can grow over several cultivations without supplementation with *myo*-inositol [1]. This LM-B was not found in *Mycobacteria*. The polar lipid and lipoglycan extracts from the complemented strain *C. glutamicum ∆ipsA*::pK18*-*ipsA grown on minimal media supplemented with glucose displayed normal levels of AcPIM_2_, PI, LM and LAM biosynthesis, with profiles identical to wild-type. These data are consistent with the view that IpsA affects an early stage of PI biosynthesis, namely the expression of *ino1*. In fact, the polar lipid and lipoglycan extracts obtained from Δ*ipsA* complemented with plasmid-encoded *ino1* also displayed lipid profiles identical to that of the wild-type, indicating involvement of IpsA in transcriptional activation of *ino1* expression. The results imply that in absence of IpsA, PI-based PIMs, LM and LAM are not synthesized as the mutant strain no longer produces the inositol monophosphate synthase required for synthesis of *myo*-inositol, a substrate for PI production. The polar lipid extracts of *C. glutamicum ∆ipsA* complemented with the homolog from *C. diphtheriae* exhibited a profile akin to wild-type. Thus DIP1969 is a functional equivalent of IpsA and can be designated as transcriptional activator of *ino1* (DIP0115) in this pathogenic species. For the homolog of Rv3575 the situation is not as clear as for DIP1969. Although we see a positive effect on growth and a partial complementation in terms of mycothiol biosynthesis and morphological phenotype, no PI derived lipids could be identified on the TLC results. One possible explanation for this is that most of the inositol formed is channeled towards mycothiol. Consequently, a low amount of PI derived lipids might be formed, sufficient for partial complementation of the phenotype but still too low for detection by TLC. Furthermore, the differences in *M. tuberculosis* IpsA structure, binding affinity and specificity can also be a reason for the only partial complementation. The fact that we did observe partial complementation with respect to most phenotypes tested hints at least to a very similar function in *M. tuberculosis*.

## Conclusions

In this study, we have characterized the LacI regulator IpsA as a first regulator of inositol anabolism in the *Corynebacteriales.* The findings provide insights into the regulatory mechanisms involved in processes that have been identified as promising targets for drug development. IpsA itself could be such a target, if a *myo-*inositol analog could be developed that blocks activation of *ino1*; it is noteworthy that a trehalose analog with a bacteriostatic effect on *Mycobacterium aurum* has been reported [34]*.* Finally, the IpsA regulon delivers a promising starting point for the analysis of further, so far unstudied players in cell wall synthesis in this important class of bacteria.

## Methods

### Bacterial strains, plasmids and growth media

The bacterial strains and plasmids used in this study are listed in Additional file 1: Table S1. The *C. glutamicum* type strain ATCC 13032 was used as wild-type. Growth experiments were performed at 30°C and 1,200 rpm in a Biolector system (m2p-labs, Baesweiler, Germany) in 48-well FlowerPlates containing 750 μL CGXII minimal medium [35] supplemented with 3,4-dihydroxybenzoate (30 mg/L) and carbon source as indicated. If appropriate, 25 μg/mL kanamycin was added. All cloning was performed in *Escherichia coli* DH5α cultivated at 37°C in lysogeny broth (LB, [36]) with 50 μg/L kanamycin.

### Recombinant DNA work

Routine methods such as polymerase chain reaction (PCR), DNA restriction and ligation were performed using standard protocols [36-38]. The oligonucleotides used in this study were obtained from Eurofins MWG Operon (Ebersberg, Germany) and are listed in Additional file 1: Table S2. DNA sequencing was performed by Eurofins MWG Operon (Ebersberg, Germany). The Δ*ipsA* mutant of *C. glutamicum* was constructed via a two-step homologous recombination protocol as described previously [39]. For further details regarding plasmid and mutant construction, see Additional file 1.

### DNA microarrays

For transcriptome analysis, *C. glutamicum* wild-type and Δ*ipsA* cells were grown in 5 mL BHI (brain-heart infusion, Becton, Dickinson and Company, Le Pont de Claix, France) for about 6 h at 30°C. A second precultivation was performed in CGXII minimal medium containing 2% (w/v) glucose as carbon source. The main cultures were inoculated to an OD of 0.5 in CGXII minimal medium with 2% (w/v) glucose. At an OD of 5 the cells were harvested by centrifugation (4,120 *g*, 10 minutes and 4°C). The cell pellet was subsequently frozen in liquid nitrogen and stored at -70°C. The preparation of total RNA was performed as described previously with the RNeasy Kit from Qiagen (Hilden, Germany) [40]. Synthesis of fluorescently-labeled cDNA was carried out as described in [40,41]. Purified cDNA samples to be compared were pooled and the prepared two-color samples were hybridized at 65°C while rotating for 17 h using Agilent’s Gene Expression Hybridization Kit (Agilent, Böbligen, Germany), hybridization oven and hybridization chamber. After hybridization, the arrays were washed using Agilent’s Wash Buffer Kit according to the manufacturer’s instructions. Fluorescence of hybridized DNA microarrays was determined at 532 nm (Cy3) and 635 nm (Cy5) at 5 μm resolution with a GenePix 4000B laser scanner and GenePix Pro 6.0 software (Molecular Devices, Sunnyvale, CA, USA). Fluorescence images were saved to raw data files in TIFF format (GenePix Pro 6.0). Quantitative TIFF image analysis was carried out using GenePix image analysis software and results were saved as GPR-file (GenePix Pro 6.0). For background correction of spot intensities, ratio calculation and ratio normalization, GPR-files were processed using the BioConductor R-packages limma and marray [42]. Array data were deposited in the GEO database [43] under accession number GSE50210.

### Overproduction and purification of IpsA

*E. coli* BL21(DE3) carrying the expression plasmids pET-TEV-ipsA or pAN6-ipsA-STREP were grown in LB medium at 37°C and 120 rpm. IpsA overproduction was induced by addition of 50 μM isopropyl β-d-1-thiogalactopyranoside (IPTG) followed by cultivation at 16°C for 20 h before the cells were harvested by centrifugation. Nickel chelate and StrepTactin affinity chromatography were performed as described previously [44,45]. For further purification and determination of the molecular weight, gel filtration was performed using a Superdex™ 200 10/300 GL column (GE Healthcare, Munich, Germany) (buffer: 50 mM Tris-HCl pH 8, 250 mM NaCl, 1 mM dithiothreitol (DTT)). Note that the IpsA-variant with the C-terminal STREP-tag formed less aggregates than the variant with the His_10_-tag and eluted at a volume corresponding to 79 kDa, suggesting dimer formation (Additional file 1: Figure S2). The protein was concentrated, flash frozen in liquid nitrogen and stored in the gel filtration buffer at -70°C.

### Electrophoretic mobility shift assays (EMSAs)

EMSAs were performed as described previously with the following modifications [46]. Unless otherwise stated, the STREP-tag variant was used for the EMSAs. Purified IpsA was incubated with DNA fragments (30 to 500 bp, final concentration 0.028 to 1 μM) in binding buffer (50 mM Tris-HCl pH 7.5, 40 mM KCl, 5 mM MgCl_2_) in a total volume of 10 μL. Electrophoresis was performed with 10% to 15% native polyacrylamide gels at room temperature and 150 or 180 V for 45 to 60 minutes (depending on the size of the DNA fragments). For the testing of effector molecules, IpsA was first incubated with the putative effector for 20 minutes at room temperature, followed by addition of the DNA, further 20 minutes incubation and electrophoresis as described above. As control AcnR protein and the *acn* promoter fragment was used as described in [47] (Additional file 1: Figure S7).

### Fluorescence microscopy

For recording fluorescence microscopy images, cells were centrifuged and resuspended in phosphate-buffered saline (PBS, 137 mM NaCl, 2.7 mM KCl, 4.3 mM Na_2_HPO_4_, 1.4 mM KH_2_PO_4_, pH 7.3) containing 200 ng/mL DAPI and 300 ng/mL nile red. After 5 to 10 minutes incubation at room temperature, the cells were analyzed on agar pads using a Zeiss Axioplan 2 imaging microscope that was equipped with an AxioCam MRm camera and a Plan-Apochromat 100×, 1.40 Oil DIC oil-immersion objective. Digital images were acquired and analyzed with AxioVision 4.6 software (Zeiss, Göttingen, Germany).

### Promoter fusion studies

To analyze the regulation of the *ino1* promoter by IpsA *in vivo*, a DNA fragment covering the cg3323 promoter region was fused to the *eyfp*-coding sequence (pJC1**-**Pcg3323-eYFP). Wild-type and Δ*ipsA* cells were transformed with the resulting plasmid and the fluorescence output was analyzed on minimal medium with 2% (w/v) glucose or *myo*-inositol in the Biolector system.

### Extraction and determination of mycothiol

The determination of mycothiol was carried out as described [21]. Briefly, cells were first cultivated in BHI medium for 6 h and then twice in CGXII minimal medium with 4% (w/v) as carbon source at 30°C and 120 rpm for 16 h and 24 h, respectively. 200 mg of wet cells were resuspended in 1 mL of warm (60°C) acetonitrile containing 20 mM Tris-HCl, pH 8.0 and 2 mM bromobimane (Sigma-Aldrich, Taufkirchen near Munich, Germany), sonificated for 20 s and further incubated in a 60°C water bath for 15 minutes in the dark. The samples were acidified with 5 μL of 5 M methanesulfonic acid and the cellular debris was removed by centrifugation at 16,000 ***g*** for 10 minutes. The supernatants were filtered and diluted fivefold in 10 mM methanesulfonic acid. A wild-type control sample was incubated with 5 mM NMM to block the thiol groups prior to derivatization with bromobimane. The sample were analyzed using a C_18_ column (LiChrospher RP 18, 125 × 4 mm) and the solvents A (100 mM sodium acetate buffer pH 7.2) and B (100% methanol) a flow rate of 0.7 mL/minute and the following gradient: 0 minutes, 15% B; 1 minute, 15% B; 1.5 minutes, 35% B; 11 minutes, 80% B; 12 minutes, 95% B; 13 minutes 100% B. The peak assumed to be mycothiol (5.61 minutes) was identified by comparison of the wild-type chromatogram with the one from the Δ*mshC* strain and the NMM treated sample. Furthermore this peak appears shortly after glutathione (5.14) as published for a similar method [48].

### Growth of bacteria for lipid and lipoglycan analysis

Overnight cultures of *C. glutamicum* ATCC 13032, *C. glutamicum* Δ*ipsA*, *C. glutamicum* Δ*ipsA*::pK18int-ipsA*,* and *C. glutamicum* Δ*ipsA* harboring *ino1* (cg3323), Rv3575 and DIP1969 in plasmid pAN6 were pregrown in 20 mL of 3.7% brain-heart-glucose bouillon (Sigma-Aldrich, Gillingham, UK) containing Kanamycin (25 μg/mL). The cells were washed once with PBS, used to inoculate a second preculture to an OD_600_ of 1 in 50 mL CGXII minimal medium (50 mL) supplemented with 2% glucose and incubated with gentle shaking overnight at 30°C. The cells of the second preculture were washed once with PBS and used to inoculate the main cultures to an OD_600_ of 0.1 in 10 mL CGXII minimal medium (10 mL), supplemented with either 2% glucose or 2% *myo*-inositol and grown until early log phase. At early log phase, the cells were labeled with 1 μCi/mL [1,2-^14^C]-acetate (1.66 to 2.22GBq/mmol, PerkinElmer Inc., Waltham, Massachusetts, USA) and incubated overnight at 30°C with gentle shaking. The cells were recovered following centrifugation at 3,500 ***g*** and dried.

### Analysis of polar lipids and lipoglycans

The cell cultivation procedure for the following experiments is described in the Additional file 1. Polar and apolar lipid extracts were prepared from the dried cell pellets using established procedures [49]. The dried polar lipid extracts were resuspended in CHCl_3_:CH_3_OH (100 μL, 2: 1, v/v) and the incorporation of [1,2-^14^C] acetate in lipid extracts determined by counting an aliquot (5%) using scintillation fluid (5 mL). To analyze the lipid profiles, equal counts of polar lipid extracts (20,000 cpm) were applied to silica thin layer chromatography (silica gel 60 F_254_ Merck 5554, TLC plates) and developed using the solvent system: CHCl_3_:CH_3_OH:H_2_O (60:30:6, v/v/v) in the first dimension and CHCl_3_:CH_3_CO_2_H:CH_3_OH:H_2_O (40:25:3:6, v/v/v/v) in the second dimension. The autoradiograms were obtained by exposing the TLC results to X-ray films (Kodak-Biomax MR Kodak, Rochester, New York, USA) for 48 h.

Lipoglycans were extracted using previously described protocols [50]. Briefly, dried cell pellets from a 10 mL volume culture were resuspended in water and refluxed five times with equal volume of 50% aqueous C_2_H_5_OH at 85°C, for 6 h intervals. The suspension was centrifuged and the supernatant recovered between each extraction following centrifugation. The combined supernatant fractions were dried and subjected to hot phenol-H_2_O treatment at 65°C. The aqueous phase containing the crude lipoglycan fraction was recovered and dialyzed (molecular weight cut-off 3.5 KDa) against water. The dialyzed fraction was dried and 20 μg of lipoglycans loaded on a 15% SDS-PAGE gel. The lipoglycans were visualized using Pro-Q emerald glycoprotein stain (Life Technologies, Darmstadt, Germany).

## Abbreviations

BHI: brain-heart infusion; CDP-DAG: cytidine diphosphate-diacylglycerol; DAG: diacyl-glycerol; eyfp: enhanced yellow fluorescent protein; GL-A: GlcAGroAc_2_; 1,2-di-*O*-C_16_/C_18:1_-(α-d-glucopyranosyluronic acid)-(1→3)-glycerol; GL-X: ManGlcAGroAc_2_; 1,2-di-*O-*C_16_/C_18:1_-(α-d-mannopyranosyl)-(1→4)-(α-d-glucopyranosyluronic acid)-(1→3)-glycerol; IMP: inositol monophosphatase; LAM: liporarabinomannan; LM: lipomannan; MSH: mycothiol; PBS: phosphate-buffered saline; PI: phosphatidylinositol; PIMs: PI mannosides.

## Competing interests

The authors declare that they have no competing interests.

## Authors’ contributions

MB designed and coordinated the study, constructed the mutant, performed the microarray experiments, the mycothiol measurements and fluorescence microscopy and prepared the figures. KL and CG carried out the recombinant DNA work, the electrophoretic mobility shift assays (EMSAs), the protein purification, the promoter fusion studies and the growth experiments. SG and GSB performed and analyzed the cell wall lipid studies and prepared the respective parts of the manuscript. MB and JF conceived of the study and wrote the manuscript. All authors read and approved the final manuscript.

## Supplementary Material

Additional file 1 Additional methods, figures and tables. Click here for file
