# Supplementary material for: IpsA, a novel LacI-type regulator, is required for inositol-derived lipid formation in Corynebacteria and Mycobacteria
Source: BMC Biol. 2013 Dec 30;11:122. doi: 10.1186/1741-7007-11-122 (PMC3899939; doi:10.1186/1741-7007-11-122)
Supplement: Additional file 1 — Additional methods, figures and tables. [file 1741-7007-11-122-S1.pdf]

Supplemental Material
to

"IpsA, a novel lacI-type regulator, is required for inositol-derived lipid formation in Corynebacteria and Mycobacteria", by Meike Baumgart et al

Supplemental methods

Growth experiments. For growth experiments, 5 ml of brain heart infusion broth (BHI, Difco Laboratories, Detroit, MI, USA) was inoculated with a colony from a fresh BHI agar plate and incubated for 6-8 h at 30°C and 220 rpm. Cells from this preculture were washed once in phosphate buffered saline (PBS, 137 mM NaCl, 2.7 mM KCl, 4.3 mM Na_2HPO_4, 1.4 mM KH_2PO_4, pH 7.3) and used to inoculate a second preculture, consisting of 20 ml CGXII minimal medium [1] supplemented with 3,4-dihydroxybenzoate (30 mg l^{-1}) as an iron chelator and 2 % (w v^{-1}) glucose as a carbon source, to an OD_{600} of about 1. For sequential cultivations, cells from the stationary phase were diluted in fresh medium to a starting OD_{600} of 1. After overnight incubation at 30°C and 120 rpm the main cultures were inoculated to an OD_{600} of about 1 and incubated at 30°C and 1200 rpm in a Biolector (m2p-labs, Baesweiler, Germany) in 48-well FlowerPlates containing 750 µl CGXII minimal medium and different carbon sources, as specified in the text. For induction of the expression of genes under the control of the P_{tac}-promoter, isopropyl *β*-D-1-thiogalactopyranoside (IPTG) was used at the concentrations specified. Where appropriate, the medium was supplemented with 25 µg l^{-1} Kanamycin.

Recombinant DNA work

For the construction of the deletion plasmid, the up- and downstream regions (~500 bp) of *ipsA* were amplified using the oligonucleotide pairs Delta_cg2910_1(XmaI)/Delta_cg2910_2 and Delta_cg2910_3/Delta_cg2910_4(XbaI), respectively. The resulting PCR products served as templates for overlap extension PCR using the oligonucleotide pair Delta_cg2910_1(XmaI)/ Delta_cg2910_4(XbaI). The resulting DNA fragment was digested with XmaI and XbaI and cloned into pK19*mobsacB*. Transfer of the sequenced plasmid pK19*mobsacB*-ΔipsA into *C. glutamicum* and screening for the first and second recombination event were performed as described [2]. Kanamycin-sensitive and sucrose-resistant clones were tested by colony PCR analysis with the oligonucleotide pair Delta_cg2910_for/Delta_cg2910_rev for the deletion of cg2910.

For the construction of pET-TEV-ipsA, pAN6-ipsA and pAN6-ipsA-STREP, the *ipsA*-coding region was amplified using the primer pairs cg2910-NdeI-fw/cg2910-EcoRI-rv (for pET-TEV-ipsA and pAN6-ipsA) and cg2910-NdeI-fw/cg2910-NheI-rv-nostop (for pAN6-ipsA-STREP) and chromosomal DNA of *C. glutamicum* as template. The PCR products were cut with the enzymes given in the oligoname and ligated into pET-TEV and pAN6 cut with the same enzymes.

For plasmid pAN6-cg3323, the cg3323 coding region was amplified using the primer pair cg3323-NdeI-fw/cg3323-NheI-rv and chromosomal DNA of *C. glutamicum* as template. The PCR product was cut with the enzymes given in the oligoname and ligated into pAN6 cut with the same enzymes.

For plasmid pJC1-venus-term, the venus coding region was amplified using the primer pair eYFP-Bam-NdeI-fw/Venus-term2-rv and plasmid DNA of pXVENC-2 as template. The terminator-encoding region was amplified using the primer pair Venus-term3-fw/Term4-SalI-

rv and plasmid DNA of pAN6 as template. The fragments were joined by overlap extension PCR, cut with BamHI and SalI and ligated into pJC1 cut with the same enzymes.
For plasmid pJC1-Pcg3323-eYFP, the promoter region of cg3323 was amplified using the primer pair Promcg3323-rv-YFP/Promcg3323-fw-BamHI and chromosomal DNA of *C. glutamicum* as template. The *eyfp*-coding sequence was amplified using the primer pair eYFP-SpeI-rv/eYFP-fw and plasmid DNA of pEKEx2-eyfp as template. The fragments were joined by overlap extension PCR, cut with BamHI and SpeI and ligated into pJC1-venus-term cut with the same enzymes. The SpeI site is located between *venus* and the terminator, therefore *venus* is removed from the plasmid and only the *eyfp* reporter is used.

Table S1: Strains and plasmids used in this study

Strain or plasmid	Relevant characteristics	Source or Reference
M. tuberculosis		
H37Rv	wild-type laboratory strain, DNA used as PCR template	ATCC 25618
C. diphtheriae		
ATCC 27010	wild-type laboratory strain, DNA used as PCR template	DSM 44123
E. coli		
DH5α	F^- Φ80*dlac*Δ(*lacZ*)M15 Δ(*lacZYA-argF*) U169 *endA1 recA1 hsdR17* (r_K^- , m_K^+) *deoR thi-1 phoA supE44* λ^- *gyrA96 relA1*; strain used for cloning procedures	[3]
BL21(DE3)	F- *ompT hsdS*$_B$ (r_B-, m_B-) *gal dcm* (DE3); host for protein production	[4]
C. glutamicum		
ATCC13032	Biotin-auxotrophic wild type	[5]
ATCC13032 Δ*ipsA*	ATCC13032 with an in-frame deletion of cg2910	This work
ATCC13032::pK18int		This work
ATCC13032 Δ*ipsA*::pK18int		This work
ATCC13032::pK18int-*ipsA*		This work
ATCC13032 Δ*ipsA*::pK18int-*ipsA*		This work
ATCC13032 Δ*mshC*	ATCC13032 with a deletion of cg1709, defect in mycothiol biosynthesis	[6]
**Plasmids**		
pK19*mobsacB*	Kan^R.; plasmid for allelic exchange in *C. glutamicum*; (pK18 *oriV*$_{E.c.}$, *sacB*, *lacZ*α)	[7]
pK19*mobsacB*-ΔipsA	Kan^R; pK19*mobsacB* derivative containing a PCR product covering the up- and downstream regions of *ipsA* (cg2910)	This work
pK18-int1	KanR; plasmid for integration of foreign DNA into the intergenic region between cg1121-cg1122 (*oriV*$_{E.c.}$, *sacB*, *lacZ*α)	[8]
pK18int-ipsA	Kan^R; plasmid for integration of the *ipsA* enconding region including the native promoter into the intergenic region between cg1121-cg1122	This work
pEKEx2-eyfp	Kan^R, pEKEx2 containing *eyfp* with artificial RBS, under control of P_{tac}	[9]
pAN6	Kan^R; *C. glutamicum*/*E. coli* shuttle vector for regulated gene expression, derivative of pEKEx2	[10]
pAN6-ipsA	Kan^R; pAN6-derivative for expression of IpsA under control of the P_{tac} promoter	This work
pAN6-ipsA-STREP	Kan^R; pAN6-derivative for overproduction of IpsA with a C-terminal STREP-tag under control of the P_{tac} promoter	This work
pAN6-rv3575c	Kan^R; pAN6-derivative for expression of rv3575 (IpsA homolog from *M. tuberculosis*) under control of the P_{tac} promoter	This work
pAN6-DIP1969	Kan^R; pAN6-derivative for expression of DIP1969 (IpsA homolog from *C. diphtheriae*) under control of the P_{tac} promoter	This work
pAN6-cg3323	Kan^R; pAN6-derivative for expression of *ino1* under control of the P_{tac} promoter	This work
pET-TEV	Kan^R; pET28b derivative for overexpression of genes in *E. coli*, adding an N-terminal decahistdine tag and a TEV protease cleavage site to the target protein (pBR322 *oriV*$_{E.c}$., PT7, *lacI*)	[11]
pET-TEV-ipsA	Kan^R; pET-TEV derivative for overproduction of IpsA	This work

	with an N-terminal decahistidine tag which can be cleaved off using TEV protease	
pJC1	Kan^R, Amp^R; *C. glutamicum/E. coli* shuttle vector	[12]
pXVENC-2	Kan^R, *oriT oriV*, P_{xyl}, *venus*	[13]
pJC1-venus-term	Kan^R, pJC1 derivative carrying the venus coding sequence and additional terminators.	This work
pJC1-Pcg3323-eYFP	Kan^R; pJC1-venus-term derivative carrying the promoter of *ino1* (cg3323) fused to *eyfp* for promoter activity studies.	This work

Table S2: Oligonucleotides used in this study

Oligonucleotide	Sequence (5' → 3') and properties[a]
**Deletion of *ipsA* (cg2910) and PCR-analysis of the resulting mutants**	
Delta_cg2910_1(XmaI)	TATATACCCGGGAATGCGTGGATGATGCGATCATC
Delta_cg2910_2	**CCCATCCACTAAACTTAAACA**TTGTTTCCTACCCATAATCATTTC
Delta_cg2910_3	**TGTTTAAGTTTAGTGGATGGG**GGTTCCACGGTTGCGCCAATCTAG
Delta_cg2910_4(XbaI)	TATATATCTAGAATTCGTGGAGATCAAGCCTTTCC
Delta_cg2910_for	GTGATCATCATGCTCGCTGTGG
Delta_cg2910_rev	GCCAAGATTGAAGCAGATCTGG
**Construction of IpsA expression plasmid**	
cg2910-NdeI-fw	GCGCCATATGATTATGGGTAGGAAACAACAATAC
cg2910-Nhe-rv-nostop	GCGCGCTAGCGATTGGCGCAACCGTGGAACC
cg2910-EcoRI-rv	GCGCGAATTCCTAGATTGGCGCAACCGTGGAACC
**Construction and PCR-verification of the chromosomal complementation with *ipsA***	
cg2910+Prom-MfeI-fw	GCGCCAATTGAATTGGATCCGGCAGCGTTG
cg2910+Prom-XhoI-rv	GCGCCTCGAGCTAGATTGGCGCAACCGTGGA
pK18-IGR-fw	CTTGGTTCGAATATGCAGTTCGG
cg2911-fw	ATCACCCTTGGCAACGGAG
int-reg-fw	AGCACCTTCGGCAAGAAGTA
int-reg-rv	CATCGAAGGTGTCGCAAAC
M13-rv	AGCGGATAACAATTTCACACAGGA
**Construction of pAN6-cg3323**	
cg3323-NdeI-fw	GCGCCATATGAGCACGTCCACCATCAG
cg3323-NheI-rv	GCGCGCTAGCTTACGCCTCGATGATGAATGCC
**Construction of pJC1-venus-term**	
eYFP-Bam-NdeI-fw	CGCGGATCCGCGGATATCCCATATGGTGAGCAAGGGCGAGGAGCTG
Venus-term2-rv	**AAAACGACGGCCAGTACTAGTTTACTTGTACAGCTC**GTCCATGC
Venus-term3-fw	**GAGCTGTACAAGTAAACTAGTACTGGCCGTCGTTTT**
Term4-SalI-rv	ACGCGTCGACCAAAAGAGTTTGTAGAAACGCAA
**Construction of the cg3323 promoter fusion plasmid pJC1-Pcg3323-eYFP**	
Promcg3323-rv-YFP	**CTCGCCCTTGCTCACCAT**CTAAAATTTCTCCTCTTAAAAAGATAACGGC
Promcg3323-fw-BamHI	GCGCGGATCCGGAAATCTCCCGAACATCAGAAG
eYFP-SpeI-rv	GGACTAGTTTATCTAGACTTGTACAGCTCGTCCATG
eYFP-fw	ATGGTGAGCAAGGGCGAGGAG
**Oligonucleotides for the expression plasmids of IpsA homologs of *M. tuberculosis* and *C. diphtheriae***	
DIP1969-NdeI-fw	GCGCCATATGGTGGTGCCTATGGCTTCC
DIP1969-NheI-rv	GCGCGCTAGCCTAGCCTCGCCGATGCTC
Rv3575c-NdeI-fw	GCGCCATATGAGTCCCACACCGCGGAGG
Rv3575c-NheI-rv	GCGCGCTAGCTTACGCCGGCGGACCCGC
**PCR products used in gel shift assays (~500 bp fragments)**	
cg0044-fw	GCAGATTGCAACATCGTGGAC
cg0044-rv	GATTAACAGCGCAGCACCAATG
cg0043-fw	TAGTGATGCCGTGGCTACTC
cg0043-rv	GCGATTTCTGGCACAAGGTTG

cg0326-fw	GCTTCGATAAGCTCCTGGTTG
cg0326-rv	CAAGGCAAACTTGACGTCGAC
cg0404-fw	CGCAATAAGTTTCGCCTTACAGG
cg0404-rv	GGAGCTTCATCGGTGTATTTGC
cg0508-fw	CGACCACCCTCTCAACAGGTG
cg0508-rv	GAGTGCAAAAACAGTAACGGCAG
cg3405-fw	TCGATTTCACCGCTGTCGAG
cg3405-rv	CTTGAATACGAACCTGACCTGG
cg2911-fw	ATCACCCTTGGCAACGGAG
cg2911-rv	CAGTTGAGCAGCCAGCTAG
cg3323-fw	GGAAATCTCCCGAACATCAGAAG
cg3323-rv	AATGGCAACCCTGATGGTGG
cg3195-fw	TCGTCACATGGTGGTTTCCTAC
cg3195-rv	GGCCAAGGTGGTTAAGTCTTG
cg0623-fw	ACTGCTGAGGTAAACCCTGAG
cg0623-rv	CACAACCAGCAATACCCAACG
cg2896-fw	CGTTCTACGCAATCGCTGTAG
cg2896-rv	AAGCCGTCACCACGGCGGTAA
cg0625-fw	GTCGCAGGAGATAACGAAGC
cg0625-rv	CACGGTTGCAGCAGACCA
cg2870-fw	AACCGGTGGAGAATTCCTTCTG
cg2870-rv	TTGACCTCAACACTTGAAGATTCTG
cg1421-fw	CGTTATTGCTGGCGAAGGC
cg1421-rv	GATTGCGGTGCCCATGTTG
cg1055-fw	ATCATCCGAACCAGGGAAACG
cg1055-rv	AGGTCTGCGGTGGCAATG
cg0727-fw	GAAGCATTGGAAACTACTTTAGCGC
cg0727-rv	CTCTTGGGTCTTTAGTGAATCGAG
cg3389-fw	GAGCTATTCCAACACTTGGACG
cg3389-rv	CGGGTGGGTGATGCCTAG
cg0534-fw	GCTGGTGGTGCGTTATGATTC
cg0534-rv	CCAAGAGGATACGTGCGATG
cg2181-fw	CACGCGAATTAACGCTTATCGAC
cg2181-rv	GGTTACAGCGAGAGACTTCTTC
cg1918-fw	ATAAGCATCAGAGCCATGCTCG
cg1918-rv	GTTGCGGGTGAGGGTCTGA
cg3138-fw	GCAGTCGATGAGAACAGGAATC
cg3138-rv	TCCAGGAGTGCGCTCTTCTA
cg1290-fw	AACCACACGTCACCGCGTTGC
cg1290-rv	AGCGACAGTGGAAGAAAAGTTGG
cg2061-fw	AGCTTGATGATGTAGAAGCGAAAG
cg2061-rv	GACGCCTGCAGCCTTGGA
cg0936-fw	CCGGAAAGTTACATCGCTACC
cg0936-rv	GGAGCTAGTCTTAGTGGAGTG
cg3107-fw	CTATCTAGTAGGTACGGCGC
cg3107-rv	GGTAAATTCTTGGGGTGCAGC
cg3286-fw	CGGTGTGCGGTCAGCCAT
cg3286-rv	CGTGAGGGCGAGGGTAAG
cg1580-fw	GCTTGTGGCGACTCTGAG
cg1580-rv	CATAGTTACACCATACACGTTATGC
cg0753-fw	ACCAAGCAGACAAGCTAGTACAG

cg0753-rv	GTGGTAGCAAAAGCGCCAG
cg1476-fw	GCTCTAACAACCGCCAAAAGAAGAAC
cg1476-rv	CGGGTGGATCTCATTTTGGG
cg0998-fw	TGGATTGGAAACTGCTGGGC
cg0998-rv	CGGGTTCTCACCGTTGTTTG
cg2906-fw	GCTGCGATTGCTGCAACAG
cg2906-rv	GGACGAGTGTCCGTGATTTTG
cg2909-fw	CGTGGAAATGACAGATTCCACC
cg2909-rv	GTCTTGGCGATTTCCTCAATAGTC

PCR products and oligonucleotides used for the determination of the IpsA binding sites in the cg3323 promoter

cg3323-3	GAATGGTATGTCCGTACCCTG
cg3323-4	CAGGGTACGGACATACCATTC
cg3323-5	CGTTCCAAAATGTGGGGATTCC
cg3323-6	GGAATCCCCACATTTTGGAACG
cg3323-7	CATTACCCCCATTCGGGAGTG
cg3323-8	CACTCCCGAATGGGGGTAATG
cg3323-30er-A-fw	AATGGGGGTAATGCTTGATCGATCAATTGA
cg3323-30er-A-rv	TCAATTGATCGATCAAGCATTACCCCCATT
cg3323-30er-B-fw	TGCTTGATCGATCAATTGAGTTGCTTGATCGATCAGG
cg3323-30er-B-rv	CCTGATCGATCAAGCAACTCAATTGATCGATCAAGCA
cg3323-30er-C-fw	GTTGCTTGATCGATCAGGTCTGATTTCTGC
cg3323-30er-C-rv	GCAGAAATCAGACCTGATCGATCAAGCAAC
cg3323-30er-D-fw	AGGTCTGATTTCTGCTGGGAATCCCCACAT
cg3323-30er-D-rv	ATGTGGGGATTCCCAGCAGAAATCAGACCT

PCR products and oligonucleotides used for the determination of the IpsA binding site in the cg0044 promoter

cg0044-3	GTGGTTCCTTGGTTGCGTTG
cg0044-4	CAACGCAACCAAGGAACCAC
cg0044-5	CACAGCGCAAAGCCACTGAATC
cg0044-6	GATTCAGTGGCTTTGCGCTGTG
cg0044-7	GCAGATCAGATTATCGCCTTGGA
cg0044-8	ACGATCTTGATCAAGCACATCAAGC
cg0044-9	GCTTGATGTGCTTGATCAAGATCGT
cg0044-10	CAAGTCAACGCAGGTCAGAG
cg0044-11	CTCTGACCTGCGTTGACTTG
cg0044-30er-E-fw	AGCCACTGAATCAATAAAGAAGCGTTAATA
cg0044-30er-E-rv	TATTAACGCTTCTTTATTGATTCAGTGGCT
cg0044-30er-F-fw	AAAGAAGCGTTAATAAAGTTTGACTTGTGC
cg0044-30er-F-rv	GCACAAGTCAAACTTTATTAACGCTTCTTT
cg0044-30er-G-fw	AAGTTTGACTTGTGCCTCTGACCTGCGTTG
cg0044-30er-G-rv	CAACGCAGGTCAGAGGCACAAGTCAAACTT

PCR products and oligonucleotides used for the investigation of putative IpsA binding sites in other promoters

cg3195-3	GATGGATCCTGTGGTTGAACC
cg3195-4	GGTTCAACCACAGGATCCATC
cg2896-3	CTAGTAAGCAACCCACCAAGC

cg2896-4	GCTTGGTGGGTTGCTTACTAG
cg3195-5	AATCTGTGCACCGTGGGTAC
cg3195-6	GTACCCACGGTGCACAGATT
cg3195-7	CCGTTTGTAATTCTTGCAAAGTGGG
cg3195-8	CCCACTTTGCAAGAATTACAAACGG
cg3389-3	CAATGCTCAGAGGGGTTACC
cg3389-4	GGTAACCCCTCTGAGCATTG
cg3210-30er-fw	GCTCACTTCTTGATTGATGCGGTGGCTTTT
cg3210-30er-rv	AAAAGCCACCGCATCAATCAAGAAGTGAGC
cg3195-30er-A-fw	TTCAACCACAGGATCCATCCAGTTTTCCGT
cg3195-30er-A-rv	ACGGAAAACTGGATGGATCCTGTGGTTGAA
cg1421-3	GACTTTTAGCAGCTCAACGGC
cg1421-4	GCCGTTGAGCTGCTAAAAGTC
cg0534-3	CTCGATTGCCAGGGTTCCAAC
cg0534-4	GTTGGAACCCTGGCAATCGAG
cg1918-3	CTACTGCGTCGTGTCCAC
cg1918-4	GTGGACACGACGCAGTAG
cg1918-30er-fw	TTTAATGACTTTAGCTATACTTCTATCTTG
cg1918-30er-rv	CAAGATAGAAGTATAGCTAAAGTCATTAAA
cg0534-30er-fw	CATTCTAGCTTTAGTGACCATGTCAACTAC
cg0534-30er-rv	GTAGTTGACATGGTCACTAAAGCTAGAATG
cg1421-30er-A-fw	ACTAATTACTTGACACGTCAAGTAATTAGG
cg1421-30er-A-rv	CCTAATTACTTGACGTGTCAAGTAATTAGT
cg1421-30er-B-fw	GTTGTGTTCATGATCAAAGAACTGCTCAAC
cg1421-30er-B-rv	GTTGAGCAGTTCTTTGATCATGAACACAAC
cg3195-30er-B-fw	CCAATTCATTCGATAGATCCTCGCAAAAAG
cg3195-30er-B-rv	CTTTTTGCGAGGATCTATCGAATGAATTGG
cg0534-30er-B-fw	GCTGAGCTGCTTCCAGATCCAGTTTCTGAG
cg0534-30er-B-rv	CTCAGAAACTGGATCTGGAAGCAGCTCAGC
cg0534-30er-C-fw	TTATCAAACTTTCCCGGCTGAGCTGCTTCC
cg0534-30er-C-rv	GGAAGCAGCTCAGCCGGGAAAGTTTGATAA
cg0534-30er-D-fw	CCCCAATAGTTGACACGGAAACTAATTCAT
cg0534-30er-D-rv	ATGAATTAGTTTCCGTGTCAACTATTGGGG

Oligonucleotides for the mutational analysis of the DNA binding site in the cg3323 promoter

cg3323-WT-fw	TTGAGTTGCTTGATCGATCAGGTCTGATTT
cg3323-WT-rv	AAATCAGACCTGATCGATCAAGCAACTCAA
cg3323-M1-fw	TTGAGTGTATTGATCGATCAGGTCTGATTT
cg3323-M1-rv	AAATCAGACCTGATCGATCAATACACTCAA
cg3323-M2-fw	TTGAGTTGCGGTATCGATCAGGTCTGATTT
cg3323-M2-rv	AAATCAGACCTGATCGATACCGCAACTCAA
cg3323-M3-fw	TTGAGTTGCTTGCGAGATCAGGTCTGATTT
cg3323-M3-rv	AAATCAGACCTGATCTCGCAAGCAACTCAA
cg3323-M4-fw	TTGAGTTGCTTGATCTCGCAGGTCTGATTT
cg3323-M4-rv	AAATCAGACCTGCGAGATCAAGCAACTCAA
cg3323-M5-fw	TTGAGTTGCTTGATCGATACTGTCTGATTT
cg3323-M5-rv	AAATCAGACAGTATCGATCAAGCAACTCAA
cg3323-M6-fw	TTGAGTTGCTTGATCGATCAGTGATGATTT
cg3323-M6-rv	AAATCATCACTGATCGATCAAGCAACTCAA

PCR products and oligonucleotides used for the determination of the IpsA binding site in promoters of genes of *C. diphtheriae* and *M. tuberculosis*

Rv0046c-P-fw	AACGCCGGGAAGGCTTGC
Rv0046c-P-rv	GGTAACGACTGGTGCTCACTCAT
DIP-0115-P-fw	CCTCAAAGTGGGGAGGCTT
DIP-0115-P-rv	GGCAACACGAATAGCAGACAC
DIP0115-30er-A-fw	TTTTGTAGCTGCATGATCCATCTGTACCGA
DIP0115-30er-A-rv	TCGGTACAGATGGATCATGCAGCTACAAAA
DIP0115-30er-B-fw	ATTTACTTAACCGATTAACCAGCAGTTTTA
DIP0115-30er-B-rv	TAAAACTGCTGGTTAATCGGTTAAGTAAAT
DIP0021-30er-A-fw	TGGGTCAACTTGATCAAGCAATTTCTCTTC
DIP0021-30er-A-rv	GAAGAGAAATTGCTTGATCAAGTTGACCCA
DIP0021-30er-B-fw	AACCCCACTGAATCGGTAAAGATGCAGGTC
DIP0021-30er-B-rv	GACCTGCATCTTTACCGATTCAGTGGGGTT
rv0483-30er-fw	GTGCGCGATGGGGTCCATGATGTGTTTGGT
rv0483-30er-rv	ACCAAACACATCATGGACCCCATCGCGCAC
Rv0047-A-fw	GAGCAAATTCGATGCGAAGACC
Rv0047-A-rv	ATTTGCGACAACATCACCGCGTC
Rv0047-B-fw	GTTGTCGTTGTCGTTGTATGTCTC
Rv0047-B-rv	TAGCCATGCATCGGTGACTC
Rv0047-30er-A-fw	CGCAGGTGACGGCACCATCAAGCACGTCAG
Rv0047-30er-A-rv	CTGACGTGCTTGATGGTGCCGTCACCTGCG
Rv0047-30er-B-fw	TGTCGCAAATATATCGAGGCGATACGATGA
Rv0047-30er-B-rv	TCATCGTATCGCCTCGATATATTTGCGACA
Rv0047-30er-C-fw	AAAAGGAGGTGACTCGATGCTGGAGCTCGC
Rv0047-30er-C-rv	GCGAGCTCCAGCATCGAGTCACCTCCTTTT
Rv0047-30er-D-fw	CTGGGTCTGTTGATCGAGTCACCGATGCAT
Rv0047-30er-D-rv	ATGCATCGGTGACTCGATCAACAGACCCAG
**PCR products for the control EMSA with AcnR**	
acn-Prom-5-for	ACATCACGCACGTACCCATTTCG
acn-Prom-3-rev	TAGTCATAGGACTTGTCGCC

[a]In some cases oligonucleotides were designed to introduce recognition sites for restriction endonucleases(recognition sites underlined). Complementary sequences used for overlap extension PCR are written in bold letters. Red letters indicate mutated bases.

Table S3: Genes more than four-fold regulated in the IpsA-deletion mutant compared to the wild type. Genes which are part of the DtxR/RipA-regulon or encoding ribosomal proteins are shaded in grey. * Target gene of IpsA which was identified by a genome-wide *in silico* search with the binding motif and does not fit into the criteria stated above. Array data were deposited in the GEO database (ncbi.nlm.nih.gov/geo) under accession number GSE50210.

Locus tag	Gene	Annotated function	Ratio	n	p-value
cg3323	*ino1*	*myo*-inositol-phosphate synthase	0.047	3	0.011
cg0044	*rbsB*	ABC transporter/periplasmic D-ribose-binding protein	0.082	3	0.004
cg2896		putative secreted protein, hypothetical endoglucanase	0.114	3	0.003
cg2181		ABC-type peptide transport system, secreted component	0.128	3	0.005
cg0446	*sdhA*	succinate dehydrogenase	0.138	3	0.003
cg0045		probable ABC transport protein, membrane component	0.138	3	0.028
cg2636	*catA1*	catechol 1,2-dioxygenase	0.143	3	0.003
cg0622		duplicated ATPase component SCO2324 of energizing module of predicted cobalamin ECF transporter	0.160	3	0.003
cg2182		ABC-type peptide transport system, permease component	0.167	3	0.022
cg1918		putative secreted protein	0.169	3	0.005
cg3138	*ppmA*	putative membrane-bound protease modulator	0.171	3	0.004
cg2910	*ipsA*	transcriptional regulator, LacI family	0.173	3	0.030
cg3195		flavin-containing monooxygenase (FMO)	0.178	3	0.010
cg0447	*sdhB*	succinate dehydrogenase	0.185	3	0.002
cg0621		substrate-specific component SCO2325 of predicted cobalamin ECF transporter	0.187	3	0.006
cg0445	*sdhC*	succinate dehydrogenase	0.189	3	0.005
cg0623		transmembrane component SCO2323 of energizing module of predicted cobalamin ECF transporter	0.194	3	0.004
cg3048	*pta*	phosphate acetyltransferase	0.202	2	0.082
cg0601	*rpsC*	30S ribosomal protein S3	0.212	3	0.007
cg3139		hypothetical protein cg3139	0.213	3	0.002
cg0599	*rpsS*	30S ribosomal protein S19	0.215	3	0.009
cg0600	*rplV*	50S ribosomal protein L22	0.216	3	0.007
cg0598	*rplB*	50S ribosomal protein L2	0.217	3	0.011
cg1487	*leuC*	isopropylmalate isomerase large subunit	0.218	3	0.035
cg0624		secreted oxidoreductase	0.219	3	0.005
cg0602	*rplP*	50S ribosomal protein L16	0.226	3	0.005
cg1290	*metE*	5-methyltetrahydropteroyltriglutamate-homocysteine methyltransferase	0.226	3	0.017
cg0594	*rplC*	50S ribosomal protein L3	0.233	2	0.085
cg0593	*rpsJ*	30S ribosomal protein S10	0.237	2	0.064
cg2061	*psp3*	putative secreted protein	0.244	3	0.000
cg0936	*rpf1*	resuscitation promoting factor	0.247	2	0.098
cg3107	*adhA*	Zn-dependent alcohol dehydrogenase	0.251	3	0.033
cg3210*		cell envelope-related transcriptional regulator	0,411	2	0,024
cg2870	*dctA*	Na+/H+-dicarboxylate symporter	3.948	3	0.004
cg3286		putative secreted protein	4.073	2	0.013
cg0527	*glyR*	transcriptional regulator of glyA	4.578	2	0.021
cg1580	*argC*	N-acetyl-gamma-glutamyl-phosphate reductase	4.798	3	0.003
cg3391	*oxiD*	myo-Inositol dehydrogenase	4.979	3	0.018
cg1421		putative dinucleotide-binding enzyme	5.214	3	0.000
cg0533	*menE*	O-succinylbenzoic acid-CoA ligase	5.467	3	0.001
cg0753		secreted protein	5.507	3	0.000
cg2797		hypothetical protein cg2797	5.517	3	0.023
cg3389	*oxiC*	myo-Inositol dehydrogenase	5.528	3	0.006
cg0471	*htaC*	secreted heme transport-associated protein	5.553	3	0.003
cg1476	*thiC*	thiamine biosynthesis protein ThiC	5.554	3	0.000
cg0534		putative integral membrane protein	5.597	3	0.000
cg0998		trypsin-like serine protease	5.798	3	0.001
cg2311		SAM-dependent methyltransferase	6.217	2	0.032
cg1120	*ripA*	transcriptional regulator of iron proteins, AraC family	6.983	3	0.005
cg3390		myo-Inositol catabolism, sugar phosphate isomerase/epimerase	7.674	3	0.012
cg0470	*htaA*	secreted heme transport-associated protein	8.972	3	0.008
cg3156	*htaD*	secreted heme transport-associated protein	9.695	3	0.002
cg2796		MMGE/PRPD family protein	11.215	3	0.021

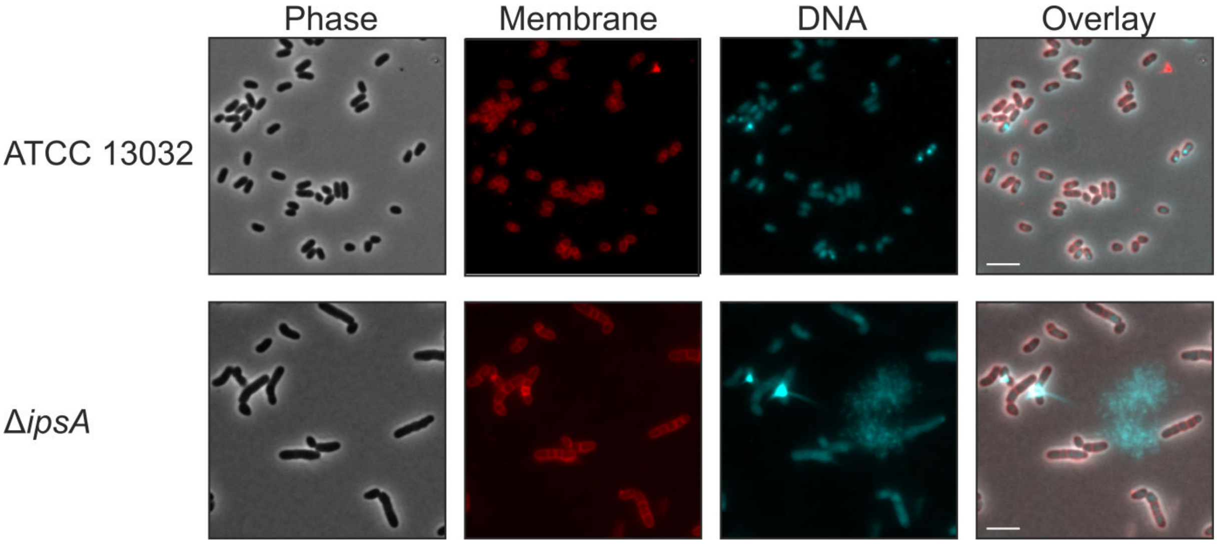

Fig. S1: Microscopic phenotype of ATCC 13032 and Δ*ipsA*. DNA was stained with DAPI (cyan) and lipophilic regions with nile red (red), scale bar 5 µm. The cells fail to divide properly, the DNA distribution is sometimes uneven and DNA can regularly be seen outside of the cells.

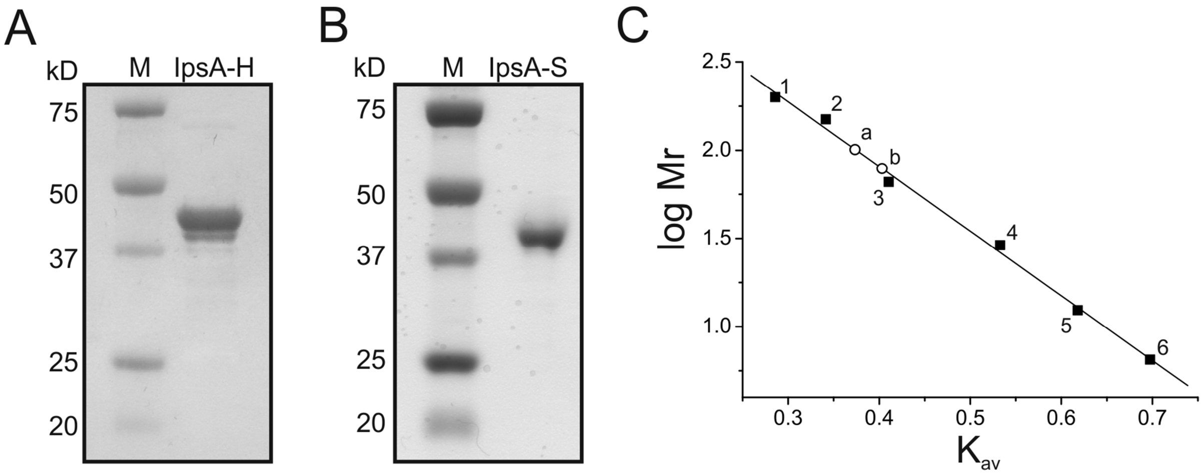

Fig. S2: Purification and determination of the molecular weight of IpsA. (A) Coomassie stained SDS-PAGE of IpsA-N-His. (B) Coomassie stained SDS-PAGE of IpsA-C-Strep. (C) Size exclusion chromatography of IpsA after purification of the His-tagged (a) or STREP-tagged (b) derivative using a Superdex 200 10/300 GL column. The standards for molecular weight determination were (1) β-amylase (200 kDa), (2) alcohol dehydrogenase (150 kDa), (3) bovine serum albumin (66 kDa), (4) carbonic anhydrase (29 kDa), (5) cytochrome C (12.4 kDa) and (6) aprotinin. IpsA eluted at a theoretical molecular weight of (a) 101 kDa or (b) 79 kDa. Gel filtration revealed less aggregated protein in the Strep-tag preparation.

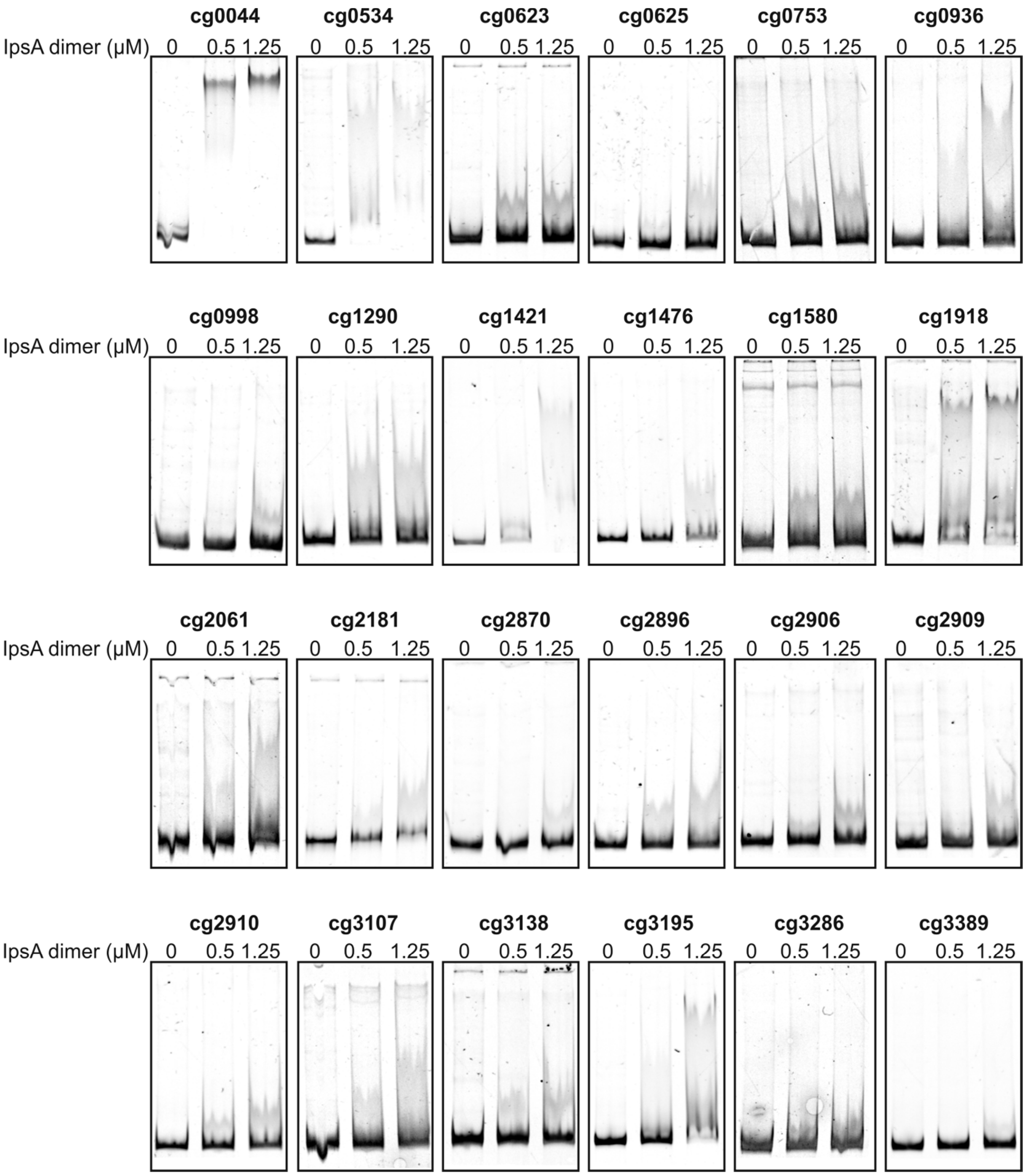

Fig. S3: Binding studies with promoter regions of putative target genes. DNA fragments of about 500 bp (90 ng) covering the respective promoter regions were incubated with IpsA-His at the given concentrations and analyzed on 10 % native polyacrylamide gels. Gels were stained with SYBR Green.

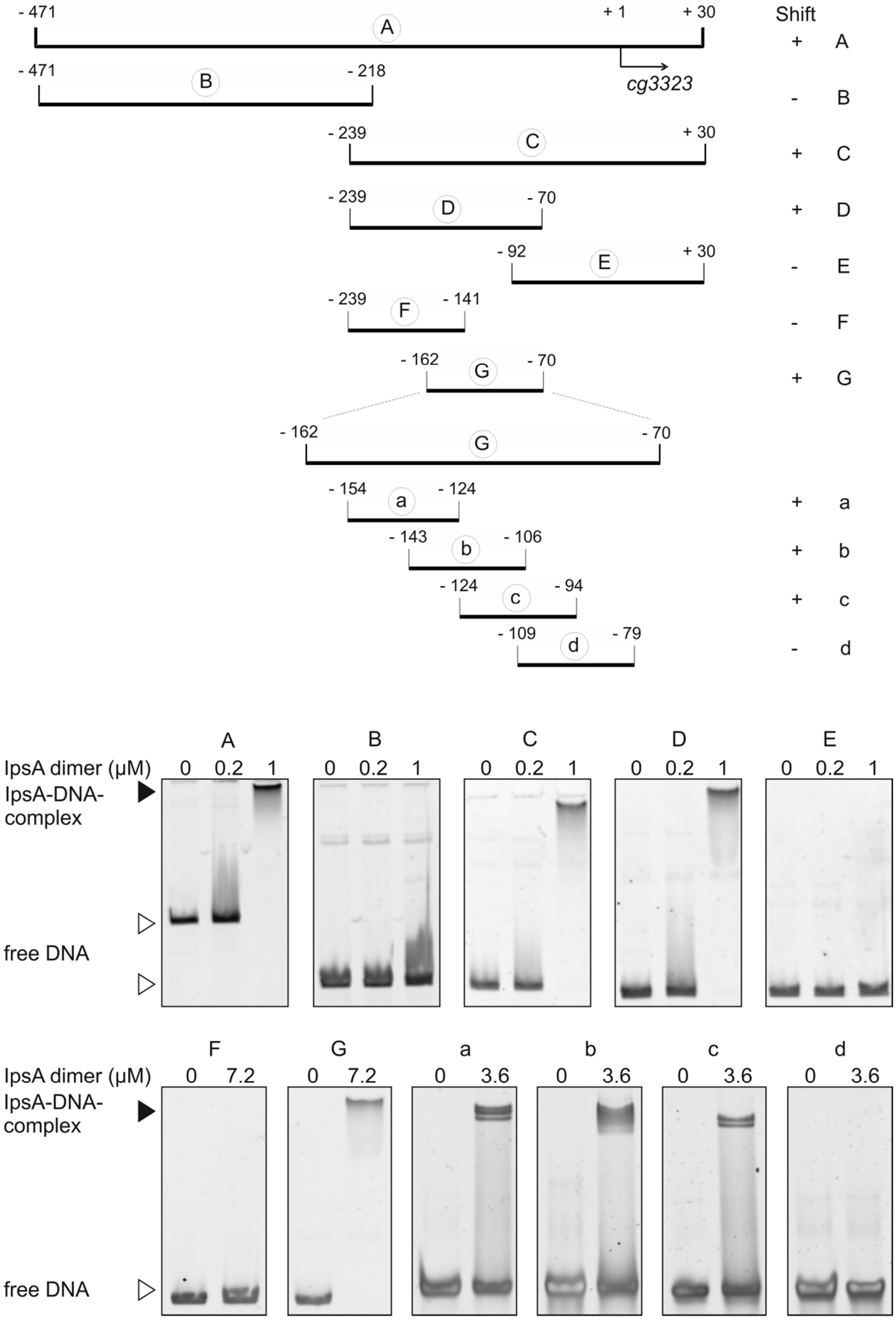

Fig. S4: Identification of the IpsA binding site in the *ino1* promoter. Several subfragments of the original 500 bp fragment were designed and tested in band shift assays for IpsA-His binding. Positions are given relative to the translational start of *ino1* (cg3323).

cg3323 GGGGTAATGCTTGATCGATCAATTGAGTTGCTTGATCGATCAGGTCTGATTTCTGC

TGGGAATCCCCACATTTTGGAACGTAGCGTCGATAAGCGTGCGG-50-bp-ATG

-35 -10 +1

cg0044 AGCCACTGAATCAATAAAGAAGCGTTAATAAAGTTTGACTTGTGCCTCTGACCTGCGTTGACTTGAGTAAATG

-35 -10 +1

cg1421 TTTCTTTTTAACTAATTACTTGACACGTCAAGTAATTAGGGTCTAGTGTTGTGTTCA-95-bp-ATG

-35 -10 +1

cg3195 GGTTCAACCACAGGATCCATCCAGTTTTCCGTCATAGGGGGTACTTTCC-90-bp-

ATGACTTGAAACACTTTATAGAGTAGAAAGTGAGTCA-24-bp-TTG

-35 -10 +1

cg3210 TAATCGCTCACTTCTTGATTGATGCGGTGGCTTTTGTGGGCTACTCCGC-94-bp-

AATACATCTGTCCCTCCTAGGCGCTACTCTATTAACCATG

-35 -10 +1

cg0534 CTCCGACGAAACCCCAATAGTTGACACGGAAACTAATTCATTCTAGCTTTAGTGACCATGTCA

-35 -10 +1

Fig. S5: Promoter regions of the IpsA target genes in *C. glutamicum*. Transcriptional start sites (red) and IpsA binding sites (blue) are given.

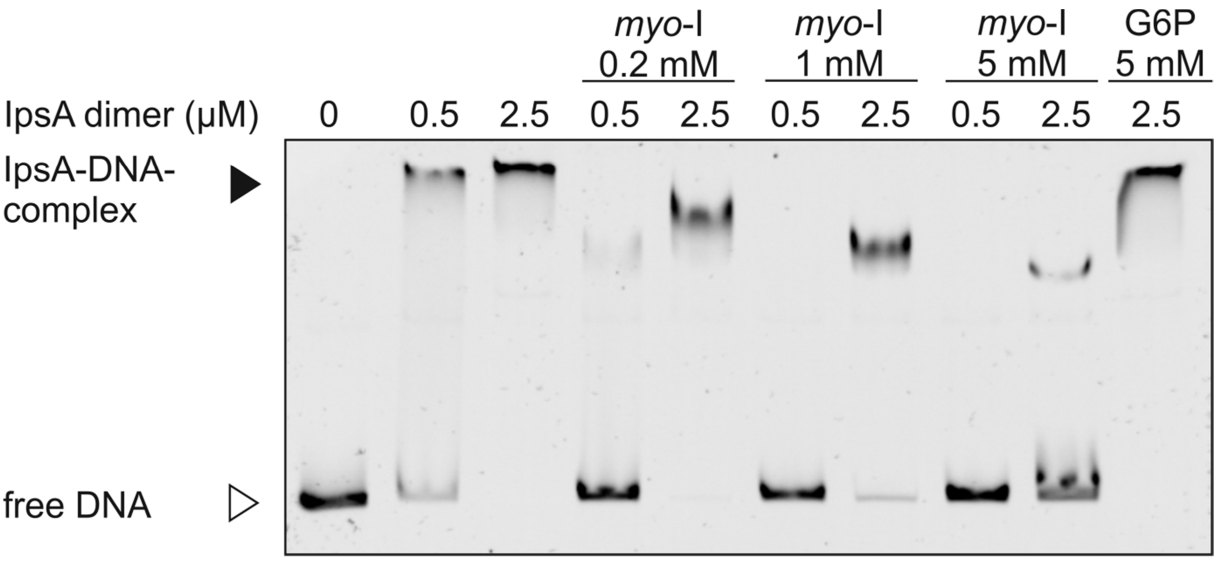

Fig. S6: Inhibition of the IpsA-DNA interaction by *myo*-inositol. A DNA fragment (169 bp) covering the promoter region of *ino1* was incubated with IpsA and *myo*-I or G6P at concentrations as indicated, and analyzed. *myo*-I, *myo*-inositol; G6P, glucose-6-phosphate.

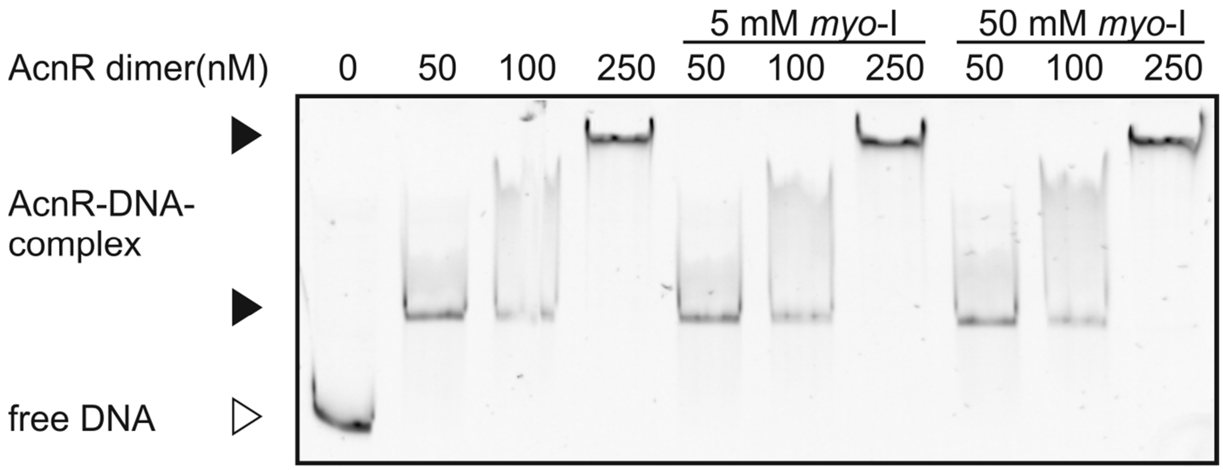

Fig. S7: Bandshift with AcnR and *myo*-Inositol. 90 ng of a 343 bp DNA fragment covering the promoter of aconitase (*acn,* cg1737) of *C. glutamicum* was incubated with AcnR protein and different concentrations of *myo*-inositol and analyzed on a 10 % native polyacrylamide gel. In the tested concentrations, *myo*-Inositol has no influence on the formation of the AcnR-DNA-complex.

```
cgb_cg0044      -TCGAAGATGTC--ATTAACCAA--CAACGCAACCAAGGAACCACAATTATTCTTGCGAC 55
cdi_DIP0021     TTCATGATAGGCGGAATAGCAGTGCCAGCAGTGCCACTGGGTCA-ACTTGATCAAGCAAT 59
                 **.:..::* *  *:**.*..:  **.*. :.***. *.. ** *.**.:**::**.*

cgb_cg0044      T-CACGATCTTGATCAAGCACATCAAGCAGATCAGATTATCGCCTTGGAAAAAGGAATCA 114
cdi_DIP0021     TTCTCTTCCTTGCTATGGTGAG--CTGCCGCTACATTGCACCCCCAG--ATGGGGGAGGA 115
                * *:* : ****.*.:.* ...  .:**.*.*...:* .:* ** :*  *:..**.*  *

cgb_cg0044      TAAAGCCACAGCGCAAAGCCACTGAATCAATAAAGAAGCGTTAATAAAGTTTGACTT-GT 173
cdi_DIP0021     ATCTGTCCCAGCTCAACCCCACTGAATCGGTAAAGATGCAGGTCAGCGGTTTGACCTCAA 175
                ::.:* *.**** ***. **********..******:**.  :.:...******* * .:

cgb_cg0044      GCCTCTGAC-CTGCGTTG-ACTTGAGTAAATGAAGCTACCAAGACTC-----GCCGCAGC 226
cdi_DIP0021     GCCGTTGATGCGACACCATGCTTGAGTGAAAAGTATAAAGAAGACCCTCAAAGCCGCCGC 235
                ***  ***  * .*.  . .*******.**:..:. :*. ***** *     *****.**

cgb_cg0044      GCTCATTGGTGCTGCGCT-GTTAAT--CACGGGTTGTTCAGCTACCGGGGGAGCACCACG 283
cdi_DIP0021     AGCCGCTGTGGCAGCCCTTGTTACTGTCACCGCATGTTCCGCTACTGGCGGTGCCCCGAG 295
                .  *. **  **:** ** ****.*  *** * :*****.***** ** **:**.**..*

cgb_cg0044      GGCAACAGATGGGGCAT 300
cdi_DIP0021     GGCTT------------ 300
                ***::


cgb_cg3323      -CACCTACTTTGTTTTC-CCCTAGAAATCCCCCAT--TTCATCACTCCCGAATGGGGGTA 56
cdi_DIP0115     TTAGCAGTAATTTTCTCGCCCTTTTGTAGCTGCATGATCCATCTGTACCGATTTACTTAA 60
                  * *:. ::* ** ** ****: :.:: *  ***  * ****: *.****:* .   :*

cgb_cg3323      ATGCTTGATCGATCAATTGAGTTGCTTGATCGATCAGGTCTGATTTCTG-CTGGGAATCC 115
cdi_DIP0115     CCGATT-AACCAGCAGTTTTAATGCTC---CACACCGG-CGGTATAGTGGCCGAGATCCA 115
                . *.** *:* * **.** :.:****    *..:*.** * *::*: ** * *.**: *.

cgb_cg3323      CCACATTTTGGAACGTAGCGTCGATAAGCGTGCGGCGAAGCTTTTTCGGTCGCGGCCGTT 175
cdi_DIP0115     CGACA-----GCACCTACC--CG-TGGGCCTCCCCCGCAGTGGGTACG-TCGTGAAGCTT 166
                * ***     *.** ** *  ** *..** * *  **.**    *:** *** *..  **

cgb_cg3323      ATCTTTTTAAGAGGAGAAATTTTAG---ATGAGCACGTCCACCATCAGGGTTGCCATTGC 232
cdi_DIP0115     ATGTTCGGTTGTTATCAAAGCCAAGGGAGTATGTGTGTCTGCTATTCGTGTTGCCATTGC 226
                ** **   ::*: .: ***   :**   .*.:* . *** .* ** .* ***********

cgb_cg3323      CGGAGTCGGAAACTGCGCGACCTCCCTCATTCAGGGTGTGGAATATTACCGAAATGCGGA 292
cdi_DIP0115     CGGTGTGGGCAACTGTGCATCGTCGCTGGTACAGGGCGTGGAGTATTACAAGGATGCGTC 286
                ***:** **.***** **.:* ** ** .*:***** *****.******....***** .

cgb_cg3323      TCCT--TCCG---- 300
cdi_DIP0115     CCCTGATCAGCAGG 300
                 ***  **.*
```

Fig. S8: Alignments of the promoter regions of cg3323 and DIP0115 as well as cg0044 and DIP0021. red: translational start site, blue: IpsA binding sites.

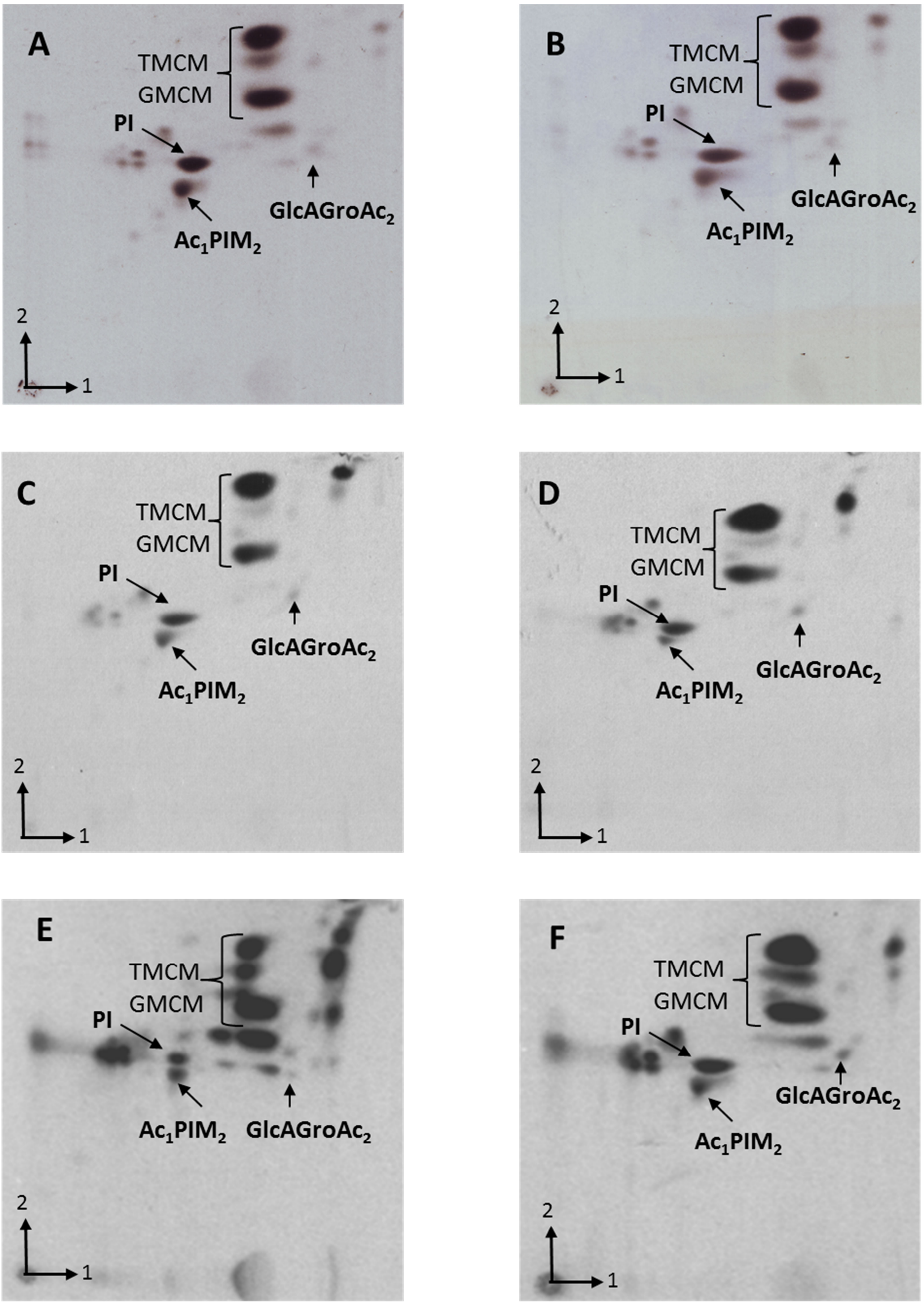

Figure S9: 2D-TLC analysis of [^{14}C]-labelled polar lipids from myo-inositol-grown cells. (A) *C. glutamicum* ATCC 13032, (B) ATCC 13032 Δ*ipsA*, (C) ATCC 13032 Δ*ipsA* pAN6-cg3323, (D) ATCC 13032 Δ*ipsA*::pK18int-ipsA, (E) ATCC 13032 Δ*ipsA* pAN6-Rv3575 and (F) ATCC 13032 Δ*ipsA* pAN6-DIP1969, cultured in CGXII supplemented with 2% (w v^{-1}) inositol. The polar lipids extracts were loaded on silica gel 60 TLCs and developed in the solvent system: $CHCl_3$:CH_3OH:H_2O (60:30:6, v/v/v) in direction 1 and $CHCl_3$:CH_3CO_2H:CH_3OH:H_2O (40:25:3:6, v/v/v/v) in direction 2.
